# Serum and nasal lavage fluid eosinophil-derived neurotoxin levels and their determinants in adults

**DOI:** 10.1016/j.jacig.2025.100510

**Published:** 2025-06-06

**Authors:** Saliha Selin Özuygur Ermis, Carina Malmhäll, Magnus P. Borres, Robert Movérare, Daniil Lisik, Reshed Abohalaka, Selin Ercan, Susanne Schmeisser, Rani Basna, Roxana Mincheva, Göran Wennergren, Jan Lötvall, Linda Ekerljung, Madeleine Rådinger, Hannu Kankaanranta, Bright I. Nwaru

**Affiliations:** a Krefting Research Centre, Institute of Medicine, Sahlgrenska Academy, University of Gothenburg, Gothenburg, Sweden; b Department of Women’s and Children’s Health, Uppsala University, Uppsala, Sweden; c Thermo Fisher Scientific, Uppsala, Sweden; d Department of Medical Sciences: Respiratory, Allergy and Sleep Research, Uppsala University, Uppsala, Sweden; e Department of Clinical Immunology, Sahlgrenska University Hospital, Gothenburg, Sweden; f Department of Clinical Science, Lund University, Lund, Sweden; g Department of Paediatrics, Sahlgrenska Academy, University of Gothenburg, Gothenburg, Sweden; h Department of Respiratory Medicine and Allergology, Sahlgrenska University Hospital, Gothenburg, Sweden; i Tampere University Respiratory Research Group, Faculty of Medicine and Health Technology, Tampere University, Tampere, Finland; j Department of Respiratory Medicine, Seinäjoki Central Hospital, Seinäjoki, Finland; k Wallenberg Centre for Molecular and Translational Medicine, University of Gothenburg, Gothenburg, Sweden

**Keywords:** Asthma, eosinophil-derived neurotoxin, eosinophil, allergy, phenotype, EDN, biomarker, adult

## Abstract

**Background:**

Eosinophil-derived neurotoxin (EDN), an eosinophil granule protein, is a candidate biomarker in asthma to reflect eosinophilic activation.

**Objective:**

We sought to characterize the distribution of serum and nasal lavage fluid (NLF) EDN levels in different adult population subgroups and assess the determinants of high EDN levels.

**Methods:**

Serum and NLF samples were collected from a population-based adult cohort study, the West Sweden Asthma Study. In total, serum EDN was measured in 2939 subjects and NLF EDN in 878 subjects, both using ImmunoCAP (Phadia AB/Thermo Fisher Scientific, Uppsala, Sweden). High EDN levels were defined as values higher than the third quartile, derived from randomly selected general adult population. Background and clinical determinants of high EDN levels were assessed in serum and NLF samples.

**Results:**

Males had higher EDN levels than females, regardless of presence of asthma or atopy. EDN levels differed by obesity and atopy, but not by age, in the random adult population. Obesity was associated with high serum EDN levels in females (adjusted odds ratio [AOR], 1.81; 95% CI, 1.08-3.04) but not in males (AOR, 1.20; 95% CI, 0.63-2.29) in the random adult population. Subjects with current asthma were more likely to have higher serum EDN levels (AOR, 1.83; 95% CI, 1.55-2.17) than those without current asthma, but not higher NLF EDN levels.

**Conclusions:**

Adults with current asthma were more likely to have higher serum, but not NLF, EDN levels than those without asthma. Although serum EDN could be a promising marker in asthma, background determinants—such as sex, obesity, and atopy—should be considered when interpreting EDN levels.

Asthma has been described as an “umbrella” term, consisting of several distinct phenotypes.^1^ Because clinical characteristics, disease course, treatment options, and response may differ between phenotypes, the identification of meaningful asthma phenotypes has become a crucial component in asthma management.^2^^,^^3^

To date, peripheral blood eosinophil count and fractional exhaled nitric oxide (Feno) have been the most commonly used biomarkers in clinical practice.^4^, ^5^, ^6^ However, current biomarkers appear to have moderate diagnostic accuracy^6^ and may have limited clinical applications, primarily because of confounding factors and stability over time.^2^^,^^7^ Moreover, combining multiple biomarkers might provide more detailed information about clinical characteristics rather than using a single marker.^8^, ^9^, ^10^, ^11^, ^12^

Although several treatment options that target eosinophilic inflammation exist, there is still an unmet requirement for reliable and more standardized biomarkers that reveal eosinophilic inflammation.^2^ Eosinophilic granule proteins are suggested as biomarkers that might reflect eosinophilic activity better and improve the accuracy of asthma phenotyping.^13^ Recently, eosinophil-derived neurotoxin (EDN), also known as ribonuclease 2 or eosinophil protein X, has gained attention because of its analytical performance, stability, and quantifiability in different samples.^13^, ^14^, ^15^ For instance, intracellular EDN concentrations of eosinophils are higher in subjects with mild allergic asthma than in healthy subjects.^16^ In addition, serum EDN levels are higher in adults with current asthma than in those who never had asthma.^17^ Some previous literature also suggest that EDN could be a marker of disease severity and may help to monitor disease course in asthma.^17^, ^18^, ^19^, ^20^, ^21^, ^22^

Although data on EDN in asthma and its clinical application seem promising,^17^, ^18^, ^19^ little is known about the factors that affect EDN levels in population-representative samples, which is crucial information for interpreting EDN levels. To our knowledge, only a few studies have previously examined EDN levels in asthma in a population setting.^17^^,^^23^^,^^24^ None of the adult studies assessed EDN levels in nasal lavage fluid (NLF). Given the paucity of data, more population-representative studies are still needed to better characterize the role of EDN in asthma. Likewise, distribution and determinants of both serum and NLF EDN levels have so far not been investigated in a representative general adult population by the recently described ImmunoCAP method.^25^

The aim of this study was to (1) characterize the distribution of serum and NLF EDN levels in adults and different subpopulations of adults, (2) assess the determinants of high serum and NLF EDN levels, and (3) determine the relation of asthma, rhinitis, and eczema to high serum and NLF EDN levels.

## Methods

### Ethics approval

Ethics approval was obtained from the Ethics Committee of the University of Gothenburg and the Swedish Ethical Review Authority (034-08, 593-08, 052/16, 906/16). The present study was performed in line with the principles of the Helsinki Declaration. A written informed consent was obtained from all participants.

### Study population

Study participants were included from the West Sweden Asthma Study,^26^ which is a population-based cohort study of adults aged 16 to 75 years residing in western Sweden. Details of the study design have been reported previously.^26^ In 2008, a postal questionnaire was sent to 30,000 randomly selected individuals, of whom 18,087 (62% response rate) participated (see Fig E1 in this article’s Online Repository at www.jaci-global.org).^27^ Of the responders, 2006 (constituting a random sample and enriched with subjects reporting asthma) took part in clinical examinations between 2009 and 2012.^26^ The participants in the 2008 questionnaire were invited for a follow-up questionnaire in 2016, from which 95 incident asthma cases were identified and clinically investigated before the start of the coronavirus disease 2019 (COVID-19) pandemic. In 2016, postal questionnaires were also sent to a new nonoverlapping random sample of adults, of whom 24,534 participated (50% response rate). A similar clinical investigation has been initiated in this second cohort, inviting a random sample, along with all subjects who reported asthma, with 953 subjects having underwent clinical investigation before COVID-19. Thus, the present study includes (1) subjects who underwent clinical examinations in the period 2009 to 2012, (2) subjects from the 2016 cohort who underwent clinical examinations before the start of COVID-19, and (3) the incident asthma cases from the first cohort (Fig E1).

### Sample selection

For serum EDN analysis, subjects with available blood samples were included in the data analysis (n = 2939) (Fig E1). NLF cell count measurement was performed only in a subpopulation of those who participated in the clinical examinations in the period 2009 to 2012, because the NLF data were available for this subpopulation; thus, EDN measurements for nasal samples were performed in those with available NLF samples and cell count measurement (n = 878) (see Table E1 and Fig E2 in this article’s Online Repository at www.jaci-global.org).

The study subpopulations were defined as follows:

1. *Random adult population* consisted of randomly selected subjects as a representative sample of the general population.
2. *Asthma sample* had subjects with current asthma.
3. *Population without asthma, any allergic disease, or atopy* consisted of subjects who did not report either of the following conditions: current asthma, current allergic rhinitis, current eczema, or atopy (defined by allergic sensitization to at least 1 positivity to skin prick test [SPT]).

### Serum and NLF EDN measurements

Serum was collected using a standardized venipuncture procedure, and nasal lavage was performed following a standard operation procedure with 5 mL of 0.9% sterile saline solution as described in detail in this article’s Online Repository at www.jaci-global.org. Serum and NLF samples of the participants were kept at −80°C in a biobank after sample collection.

Serum and NLF EDN analysis was performed using the ImmunoCAP EDN assay (research use only) (Phadia AB/Thermo Fisher Scientific, Uppsala, Sweden) according to the manufacturer’s instructions. The calibration range was 2 to 200 μg/L. Samples were diluted, when necessary, because of low sample volume or when EDN levels were higher than the upper limit of the calibration range, and the results were corrected for the dilution factor.

### High EDN levels

Serum and NLF EDN levels higher than the 75th percentile were defined as “high EDN” given the skewed distribution based on random adult population.^28^ High EDN levels were defined separately for male and female participants because of sex-related differences in EDN levels (see Fig E3 in this article’s Online Repository at www.jaci-global.org).

### Definitions of asthma, allergic diseases, and atopy

1. *Current asthma:* Self-report of physician-diagnosed/ever asthma in combination with either the presence of wheezing, asthma medication use, and attacks of shortness of breath during the last 12 months or the presence of reversibility at the clinical examination.
2. *Current rhinitis:* Positive response to any of the following questions: “Have you had problems with sneezing, runny nose, or nasal congestion without having a cold during the past 12 months?” or “Have you used medicines for hay fever/allergic nasal problems or nasal congestion of a nonallergic nature at any time during the last 12 months?” or any use of antihistamines, nasal steroid, or steroid injection.
3. *Current allergic rhinitis:* Further necessitated concomitant presence of allergic sensitization to aeroallergens defined by SPT.
4. *Chronic rhinosinusitis:* Presence of at least 2 of the following self-reported symptoms: nasal blockage; discolored nasal secretions or mucus; pain or pressure in the forehead, nose, or eyes; and impairment in smell with at least 1 positive response to nasal blockage or discolored nasal secretions/mucus for more than 12 weeks in the last 12 months.^29^
5. *Current eczema:* Positive responses to the following 2 questions: “Have you ever had an itchy rash which was coming and going for at least 6 months?” and “Have you had this itchy rash in the last 12 months?”^30^
6. *Atopy:* SPT positivity to at least 1 aeroallergen (*Dermatophagoides pteronyssinus*, *D farinae*, *Alternaria alternata*, *Cladosporium herbarum*, dog dander, cat dander, horse dander, timothy pollen, mugwort pollen, and birch pollen).

### Other background determinants

Sex, age, body mass index, family history of asthma and/or allergy, and current smoking were evaluated on the basis of questionnaire and clinical investigation.

### Statistical analysis

Because serum and NLF EDN levels showed nonnormal distribution, data were presented using median with 25th (Q1) and 75th (Q3) percentiles.^28^ In addition, the 5th to 95th percentiles (P5-P95) were presented as lower and upper limits of normal.

The Mann-Whitney *U* test was performed to compare EDN levels with categorical outcomes. The chi-square test or the Fisher test was used to compare categorical variables. Logistic regression models were used to define determinants of high EDN levels. Missing values for SPT positivity were recoded as a separate category to avoid bias in the regression models. Spearman rank correlation analysis was performed, and Spearman ρ was reported for other eosinophilic biomarkers in the random sample for NLF levels. Missing values for Feno and blood eosinophil levels were imputed using multiple imputation (see the Online Repository). Receiver-operating characteristic analysis was performed to define the area under the curve (AUC).^31^ A 2-sided *P* value was set at less than .05 for statistical significance. Data analysis was conducted using SPSS version 29.0.2.0 (IBM Corp, Armonk, NY) and R version 4.4.1 (R Foundation for Statistical Computing, Vienna, Austria). Figures were prepared by GraphPad Prism version 10.2.3 (GraphPad Software, San Diego, Calif).

### NLF EDN levels below lower limit of detection

Approximately 15% (n = 128) of the NLF samples had EDN levels below the lower limit of calibration range (<2 μg/L). In addition, 15 subjects had results less than 6 μg/L and 3 subjects had results less than 8 μg/L after correction with dilution factor. Because such values are missing not at random, they (n = 146; 17% of the NLF samples) were imputed on the basis of a Gibbs sampler approach, which has been previously suggested for left-censored data.^32^

## Results

### Background characteristics

Background characteristics for all participants, the random adult population, and subjects with current asthma are provided in Table I. The mean age was 49.8 ± 15.5 years, 57.3% were female, and 18.9% were obese among all participants. In the random adult population, 14.3% had current asthma, 27.9% had atopy, and 41.7% had asthma, any allergic disease, or atopy. Among subjects with current asthma, 50.8% had atopy. Among those with NLF EDN measurement, the mean age was 46.8 ± 15.8 years, 54.8% were female, and 18.5% were obese. In the random adult population, 15.9% had current asthma, 32.1% had atopy, and 46.5% had asthma, any allergic disease, or atopy (Table I).

**Table I:** Characteristics of study participants by serum and NLF EDN levels in all participants, random adult population, and subjects with current asthma

Characteristics	Serum EDN levels (μg/L)			NLF EDN levels (μg/L)		
	All participants (N = 2939)	Random adult population (n = 1142)	Subjects with current asthma (n = 1499)	All participants (N = 878)	Random adult population (n = 527)	Subjects with current asthma (n = 386)
**Age (y), mean ± SD**	49.8 ± 15.5	50.5 ± 15.4	49.5 ± 15.6	46.8 ± 15.8	48.6 ± 15.8	44.8 ± 15.7
**Age (y) in strata, n (%)**						
**18-30**	408 (13.9)	159 (13.9)	219 (14.6)	170 (19.4)	91 (17.3)	87 (22.5)
**31-45**	773 (26.3)	281 (24.6)	395 (26.4)	270 (30.8)	142 (26.9)	134 (34.7)
**46-60**	895 (30.5)	346 (30.3)	460 (30.7)	226 (25.7)	148 (28.1)	86 (22.3)
**61-82**	863 (29.4)	356 (31.2)	425 (28.4)	212 (24.1)	146 (27.7)	79 (20.5)
**Sex, n (%)**						
**Male**	1255 (42.7)	534 (46.8)	576 (38.4)	397 (45.2)	246 (46.7))	167 (43.3)
**Female**	1684 (57.3)	608 (53.2)	923 (61.6)	481 (54.8)	281 (53.3)	219 (56.7)
**Body mass index (kg/m^2^), n (%)**						
≤24.9	1178 (40.1)	474 (41.5)	539 (36.0)	383 (43.6)	240 (45.5)	153 (39.6)
25-29.9	1176 (40.0)	495 (43.3)	583 (38.9)	333 (37.9)	208 (39.5)	142 (36.8)
≥30	556 (18.9)	173 (15.1)	363 (24.2)	162 (18.5)	79 (15.0)	91 (23.6)
Missing data	29 (1.0)	—	14 (0.9)	—	—	—
**Smoking status, n (%)**						
***Ever smoking***						
No	1530 (52.1)	579 (50.7)	764 (51.0)	421 (47.9)	256 (48.6)	183 (47.4)
Yes	1407 (47.9)	563 (49.3)	734 (49.0)	457 (52.1)	271 (51.4)	203 (52.6)
Missing data	2 (0.1)		1 (0.1)	—	—	—
***Current smoking***						
No	2616 (89.0)	1010 (88.4)	1322 (88.2)	778 (88.6)	463 (87.9)	339 (87.8)
Yes	321 (10.9)	132 (11.6)	176 (11.7)	100 (11.4)	64 (12.1)	47 (12.2)
Missing data	2 (0.1)	—	1 (0.1)	—	—	—
**Family asthma/allergy history, n (%)**						
No	1496 (50.9)	718 (62.9)	618 (41.2)	464 (52.8)	322 (61.1)	155 (40.2)
Yes	1443 (49.1)	424 (37.1)	881 (58.8)	414 (47.2)	205 (38.9)	231 (59.8)
**Atopy, n (%)**						
No	930 (31.6)	462 (40.5)	328 (21.9)	305 (34.7)	216 (41.0)	83 (21.5)
Yes	1158 (39.4)	319 (27.9)	762 (50.8)	376 (42.8)	169 (32.1)	234 (60.6)
Missing data/nonvalid results	851 (29.0)	361 (31.6)	409 (27.3)	197 (22.4)	142 (26.9)	69 (17.9)
**Current asthma, n (%)**						
No	1390 (47.3)	978 (85.6)		491 (55.9)	442 (83.9)	
Yes	1499 (51.0)	163 (14.3)		386 (44.0)	84 (15.9)	
Missing data	50 (1.7)	1 (0.1)		1 (0.1)	1 (0.2)	
**Physician-diagnosed asthma ever, n (%)**						
No	1332 (45.3)	1003 (87.8)	146 (9.7)	471 (53.6)	454 (86.1)	40 (10.4)
Yes	1607 (54.7)	139 (12.2)	1353 (90.3)	407 (46.4)	73 (13.9)	346 (89.6)
**Current rhinitis, n (%)**						
No	743 (25.3)	525 (46.0)	165 (11.0)	289 (32.9)	227 (43.1)	59 (15.3)
Yes	2049 (69.7)	611 (53.5)	1262 (84.2)	587 (66.9)	298 (56.5)	327 (84.7)
Missing data	147 (5.0)	6 (0.5)	72 (4.8)	2 (0.2)	2 (0.4)	—
**Current allergic rhinitis, n (%)**						
No	1296 (44.1)	705 (61.7)	430 (28.7)	437 (49.8)	316 (60.0)	117 (30.3)
Yes	1018 (34.6)	246 (21.5)	712 (47.5)	322 (36.7)	133 (25.2)	217 (56.2)
**Missing data**	625 (21.3)	191 (16.7)	357 (23.8)	119 (13.6)	78 (14.8)	52 (13.5)
**Chronic rhinosinusitis, n (%)**						
**No**	2477 (84.3)	1030 (90.2)	1162 (77.5)	746 (85.0)	473 (89.8)	298 (77.2)
**Yes**	423 (14.4)	101 (8.8)	312 (20.8)	125 (14.2)	49 (9.3)	84 (21.8)
**Missing data**	39 (1.3)	11 (1.0)	25 (1.7)	7 (0.8)	5 (0.9)	4 (1.0)
**Current eczema, n (%)**						
**No**	2357 (80.2)	959 (84.0)	1128 (75.3)	708 (80.6)	441 (83.7)	290 (75.1)
**Yes**	494 (16.8)	148 (13.0)	322 (21.5)	152 (17.3)	74 (14.0)	88 (22.8)
**Missing data**	88 (3.0)	35 (3.1)	49 (3.3)	18 (2.1)	12 (2.3)	8 (2.1)
**Presence of asthma, any allergic disease, or atopy, n (%)**						
No	499 (17.0)	360 (31.5)		186 (21.2)	167 (31.7)	
Yes	2008 (68.3)	476 (41.7)		573 (65.3)	245 (46.5)	
Missing data	432 (14.7)	306 (26.8)		119 (13.6)	115 (21.8)	

Presence of asthma, any allergic disease, or atopy was defined on the basis of the presence of at least 1 of the following: current asthma, current allergic rhinitis, current eczema, or atopy (defined by at least 1 skin prick positivity to aeroallergens).

### Serum and NLF EDN levels in asthma and allergic diseases

Subjects with current asthma had significantly higher serum (median, 36.7; Q1-Q3, 24.6-56.8 μg/L vs median, 29.8; Q1-Q3, 20.7-43.4 μg/L) and NLF (median, 8.2; Q1-Q3, 3.2-17.9 μg/L vs median, 6.3; Q1-Q3, 2.8-13.7 μg/L) EDN levels than those without current asthma (Tables II and III).

**Table II:** Serum EDN levels by presence of asthma, rhinitis, eczema, and atopy in all participants

	Serum EDN levels (μg/L)				
	N/n	Median (Q1-Q3)	P5-P95	Minimum-maximum	*P* value
**All participants**	2939	33.2 (22.8-49.4)	13.7-97.1	1.0-346.5	
**Current asthma**					
**No**	1390	29.8 (20.7-43.4)	13.1-76.2	1.0-346.5	**<.001**
**Yes**	1499	36.7 (24.6-56.8)	14.7-109.0	4.6-288.6	
**Physician-diagnosed asthma ever**					
**No**	1332	29.9 (20.7-43.4)	12.9-78.9	1.0-346.5	**<.001**
**Yes**	1607	36.6 (24.7-55.4)	14.8-107.6	4.6-288.6	
**Current rhinitis**					
**No**	743	29.7 (20.7-43.1)	13.3-78.7	1.0-346.5	**<.001**
**Yes**	2049	34.6 (23.2-51.9)	13.9-101.5	4.6-288.6	
**Current allergic rhinitis**					
**No**	1296	30.8 (21.2-44.4)	13.1-81.0	1.0-346.5	**<.001**
**Yes**	1018	36.2 (24.7-54.1)	14.5-109.0	5.6-288.6	
**Chronic rhinosinusitis**					
**No**	2477	32.9 (22.8-48.3)	13.8-92.7	1.0-346.5	**.014**
**Yes**	423	35.4 (23.7-54.3)	12.9-112.8	4.6-287.7	
**Current eczema**					
**No**	2357	32.9 (22.6-48.9)	13.6-97.6	1.0-346.5	**.014**
**Yes**	494	34.9 (23.9-51.8)	14.6-94.2	7.6-230.0	
**Atopy**					
**No**	930	30.4 (20.7-43.7)	12.8-81.0	1.0-184.0	**<.001**
**Yes**	1158	35.5 (24.3-53.2)	14.5-107.0	5.6-346.5	
**Presence of asthma, any allergic disease, or atopy**					
**No**	499	28.2 (19.8-41.2)	12.3-73.3	1.0-136.0	**<.001**
**Yes**	2008	35.1 (24.1-53.4)	14.6-105.0	4.6-346.5	

The Mann-Whitney *U* test was performed to compare EDN levels. Presence of asthma, any allergic disease, or atopy was defined on the basis of presence of at least 1 of the following: current asthma, current allergic rhinitis, current eczema, or atopy (defined by at least 1 skin prick positivity to aeroallergens). Statistically significant *P* values are presented in boldface.

*P*, Percentile; *Q*, quartile.

**Table III:** NLF EDN levels by presence of asthma, rhinitis, eczema, and atopy in all participants

	NLF EDN levels (μg/L)				
	N/n	Median (Q1-Q3)	P5-P95	Minimum-maximum	*P* value
**All participants**	878	7.1 (2.9-15.9)	0-60.5	0-286.0	
**Current asthma**					
**No**	491	6.3 (2.8-13.7)	0-46.8	0-243.0	**.002**
**Yes**	386	8.2 (3.2-17.9)	0-81.5	0-286.0	
**Physician-diagnosed asthma ever**					
**No**	471	6.3 (2.7-13.7)	0-48.9	0-243.0	**.001**
**Yes**	407	8.4 (3.3-17.4)	0-74.8	0-286.0	
**Current rhinitis**					
**No**	289	5.6 (2.8-10.3)	0-32.5	0-243.0	**<.001**
**Yes**	587	8.6 (3.2-17.8)	0-69.2	0-286.0	
**Current allergic rhinitis**					
**No**	437	5.7 (2.4-11.2)	0-39.5	0-243.0	**<.001**
**Yes**	322	10.7 (5.1-23.3)	0-91.6	0-286.0	
**Chronic rhinosinusitis**					
**No**	746	6.9 (2.9-15.8)	0-53.7	0-286.0	.234
**Yes**	125	8.4 (3.0-16.5)	0-101.1	0-226.0	
**Current eczema**					
**No**	708	7.2 (3.1-15.0)	0-57.1	0-286.0	.675
**Yes**	152	6.7 (2.4-16.7)	0-74.0	0-243.0	
**Atopy**					
**No**	305	5.2 (2.1-11.2)	0-31.5	0-95.4	**<.001**
**Yes**	376	10.1 (4.5-22.4)	0-90.3	0-286.0	
**Presence of asthma, any allergic, disease, or atopy**					
**No**	186	5.5 (2.2-12.6)	0-43.3	0-95.4	**<.001**
**Yes**	573	8.1 (3.2-17.4)	0-71.2	0-286.0	

The Mann-Whitney *U* test was performed to compare EDN levels. Presence of asthma, any allergic disease, or atopy was defined on the basis of the presence of at least 1 of the following: current asthma, current allergic rhinitis, current eczema, or atopy (defined by at least 1 skin prick positivity to aeroallergens). Statistically significant *P* values are presented in boldface.

*P*, Percentile; *Q*, quartile.

Serum and NLF EDN levels were also higher in those with current rhinitis and current allergic rhinitis than in those without (Tables II and III). However, only serum EDN levels were significantly higher in those with current eczema than in those without.

Male predominance in EDN levels remained significant in subgroup analysis for those with asthma, rhinitis, and eczema and for those without asthma, any other allergic disease, or atopy (Table IV).

**Table IV:** Serum EDN and NLF EDN levels by sex in asthma, rhinitis, eczema, and atopy in all participants

	Serum EDN levels (μg/L)					NLF EDN levels (μg/L)				
	N	Median (Q1-Q3)	P5-P95	Minimum-maximum	*P* value	N	Median (Q1-Q3)	P5-P95	Minimum-maximum	*P* value
**All participants**										
**Male**	1255	37.3 (26.5-55.0)	16.6-106.0	5.6-346.5	<.001	397	9.3 (4.6-20.4)	0-67.4	0-286.0	<.001
**Female**	1684	30.2 (20.6-45.3)	12.6-84.5	1.0-288.6		481	5.7 (2.1-12.2)	0-45.8	0-226.0	
**Current asthma**										
**Male**	576	42.3 (28.6-66.9)	17.8-123.0	5.6-243.9	<.001	167	10.8 (4.9-24.3)	0-87.7	0-286.0	<.001
**Female**	923	33.6 (22.8-51.0)	13.5-97.9	4.6-288.6		219	6.7 (2.6-14.3)	0-73.7	0-226.0	
**Without current asthma**										
**Male**	655	34.6 (25.2-47.1)	14.8-86.3	6.5-346.5	<.001	230	8.8 (4.3-18.1)	0-60.1	0-243.0	<.001
**Female**	735	25.4 (18.1-37.3)	11.6-69.5	1.0-161.0		261	4.8 (0.2-10.3)	0-29.4	0-95.4	
**Physician-diagnosed asthma ever**										
**Male**	638	41.3 (28.1-65.1)	18.9-121.2	5.6-243.9	<.001	172	11.2 (5.1-23.6)	0-86.3	0-286.0	<.001
**Female**	969	33.9 (22.6-50.3)	13.5-97.2	4.6-288.6		235	6.7 (2.8-13.4)	0-68.5	0-226.0	
**Without physician-diagnosed asthma ever**										
**Male**	617	34.6 (25.1-47.2)	14.2-88.7	5.9-346.5	<.001	225	8.6 (4.1-18.0)	0-57.8	0-243.0	<.001
**Female**	715	25.7 (18.3-36.9)	11.2-71.1	1.0-216.9		246	4.8 (0-10.0)	0-33.6	0-206.0	
**Current rhinitis**										
**Male**	841	39.1 (27.1-60.0)	16.9-112.9	5.6-243.9	<.001	271	11.6 (5.2-25.0)	0-71.4	0-286.0	<.001
**Female**	1208	31.5 (21.4-47.6)	12.8-91.0	4.6-288.6		316	6.4 (2.2-13.7)	0-66.8	0-226.0	
**Without current rhinitis**										
**Male**	346	34.4 (25.4-47.1)	15.2-89.1	11.3-346.5	<.001	125	7.4 (3.7-13.8)	0-49.6	0-243.0	<.001
**Female**	397	26.3 (17.9-35.7)	12.5-71.8	1.0-178.6		164	4.1 (0.7-7.6)	0-29.0	0-147.0	
**Current allergic rhinitis**										
**Male**	458	41.7 (29.3-63.9)	17.4-120.1	5.6-243.9	<.001	161	14.2 (6.4-32.3)	2.1-89.3	0-286.0	<.001
**Female**	560	32.2 (21.9-49.2)	13.2-92.5	5.8-288.6		161	7.8 (2.9-15.0)	0-95.2	0-226.0	
**Without current allergic rhinitis**										
**Male**	524	34.4 (25.4-47.1)	16.0-87.5	5.9-346.5	<.001	180	7.5 (3.5-13.5)	0-41.9	0-243.0	<.001
**Female**	772	28.0 (19.1-40.2)	12.2-77.2	1.0-184.0		257	4.2 (0.2-9.4)	0-32.3	0-147.0	
**With chronic rhinosinusitis**										
**Male**	171	39.9 (27.9-64.4)	19.6-138.8	13.5-235.5	<.001	59	9.4 (4.7-41.7)	0-118.0	0-189.0	.005
**Female**	252	31.9 (20.7-49.8)	9.8-97.9	4.6-287.7		66	6.2 (2.0-12.6)	0-83.9	0-226.0	
**Without chronic rhinosinusitis**										
**Male**	1066	36.8 (26.2-54.0)	15.8-101.0	5.6-346.5	<.001	334	9.1 (4.5-19.6)	0-59.9	0-286.0	<.001
**Female**	1411	29.8 (20.6-44.4)	12.9-80.9	1.0-288.6		412	5.7 (2.2-12.1)	0-46.6	0-222.0	
**Current eczema**										
**Male**	171	41.4 (29.2-54.7)	19.2-96.6	12.1-230.0	<.001	54	9.2 (3.6-22.7)	0-97.5	0-243.0	.030
**Female**	323	32.5 (22.4-48.4)	13.5-91.3	7.6-216.9		98	6.5 (2.2-14.2)	0-63.8	0-226.0	
**Without current eczema**										
**Male**	1054	36.9 (25.8-55.1)	15.7-107.0	5.6-346.5	<.001	336	9.2 (4.8-19.6)	0-62.2	0-286.0	<.001
**Female**	1303	29.3 (20.2-44.4)	12.5-83.1	1.0-288.6		372	5.6 (2.1-11.8)	0-45.0	0-222.0	
**Atopy**										
**Male**	526	40.9 (28.3-61.9)	17.2-117.0	5.6-346.5	<.001	189	12.8 (6.2-27.3)	2.1-87.1	0-286.0	<.001
**Female**	632	31.8 (21.8-47.9)	13.5-89.1	5.6-288.6		187	7.4 (2.9-14.4)	0-94.9	0-226.0	
**Without atopy**										
**Male**	330	33.8 (25.2-45.8)	15.4-81.3	5.9-136.0	<.001	116	7.4 (3.1-13.3)	0-40.8	0-60.7	.001
**Female**	600	28.0 (19.0-42.0)	12.1-81.0	1.0-184.0		189	4.1 (0-9.5)	0-28.3	0-95.4	
**With asthma, any allergic disease, or atopy**										
**Male**	824	40.5 (28.1-62.8)	18.0-115.0	5.6-346.5	<.001	268	10.6 (5.1-23.0)	0-74.8	0-286.0	<.001
**Female**	1184	32.2 (22.1-48.7)	13.3-92.0	4.6-288.6		305	6.3 (2.4-12.8)	0-67.7	0-226.0	
**Without asthma, any allergic disease, or atopy**										
**Male**	198	33.6 (25.0-44.3)	14.5-81.0	7.9-136.0	<.001	71	7.5 (3.0-14.1)	0-48.4	0-60.7	.007
**Female**	301	25.1 (17.2-37.7)	11.1-71.3	1.0-122.0		115	4.6 (0-10.3)	0-34.7	0-95.4	

The Mann-Whitney *U* test was performed to compare EDN levels in male and female participants within each category. Presence of asthma, any allergic disease, or atopy was defined on the basis of the presence of at least 1 of the following: current asthma, current allergic rhinitis, current eczema, or atopy (defined by at least 1 skin prick positivity to aeroallergens).

*P*, Percentile; *Q*, quartile.

Lastly, we calculated AUC to distinguish current asthma from those without current asthma. Both serum and NLF EDN levels displayed poor discrimination (AUC, 0.611; 95% CI, 0.59-0.63 and AUC, 0.560; 95% CI, 0.52-0.60, respectively) (see Figs E4 and E5 in this article’s Online Repository at www.jaci-global.org). Sex-stratified receiver-operating characteristic curves are presented in Figs E6 to E9 (in the Online Repository available at www.jaci-global.org).

Distribution of serum and NLF EDN levels in the random adult population in relation to asthma, rhinitis, and eczema is provided in Tables E2 and E3 (in the Online Repository available at www.jaci-global.org).

### Serum and NLF EDN levels in male and female participants

Serum and NLF EDN levels were significantly higher in male than in female study participants (median of serum EDN, 37.3; Q1-Q3, 26.5-55.0 μg/L vs median, 30.2; Q1-Q3, 20.6-45.3 μg/L) (median of NLF EDN, 9.3; Q1-Q3, 4.6-20.4 μg/L vs median, 5.7; Q1-Q3, 2.1-12.2 μg/L) (Table IV). The same trend was observed in other biomarker levels because male participants had higher blood eosinophil count and Feno levels compared with female participants (see Table E4 in this article’s Online Repository at www.jaci-global.org).

### High EDN levels for serum and NLF samples

The median serum EDN level was 28.9 μg/L (Q1-Q3, 20.4-41.9), whereas the median NLF EDN level was 6.6 μg/L (Q1-Q3, 2.8-14.2) in the random adult population (Fig E3). High serum EDN values (>75th percentile) were determined as more than 41.9 μg/L for all participants, more than 46.6 μg/L for male participants, and more than 34.7 μg/L for female participants on the basis of the random adult population. High NLF EDN values (>75th percentile) were determined as more than 14.2 μg/L for the whole population, more than 18.3 μg/L for male participants, and more than 10.5 μg/L for female participants (Fig E3).

### Demographic determinants of high EDN levels

**Fig 1:**
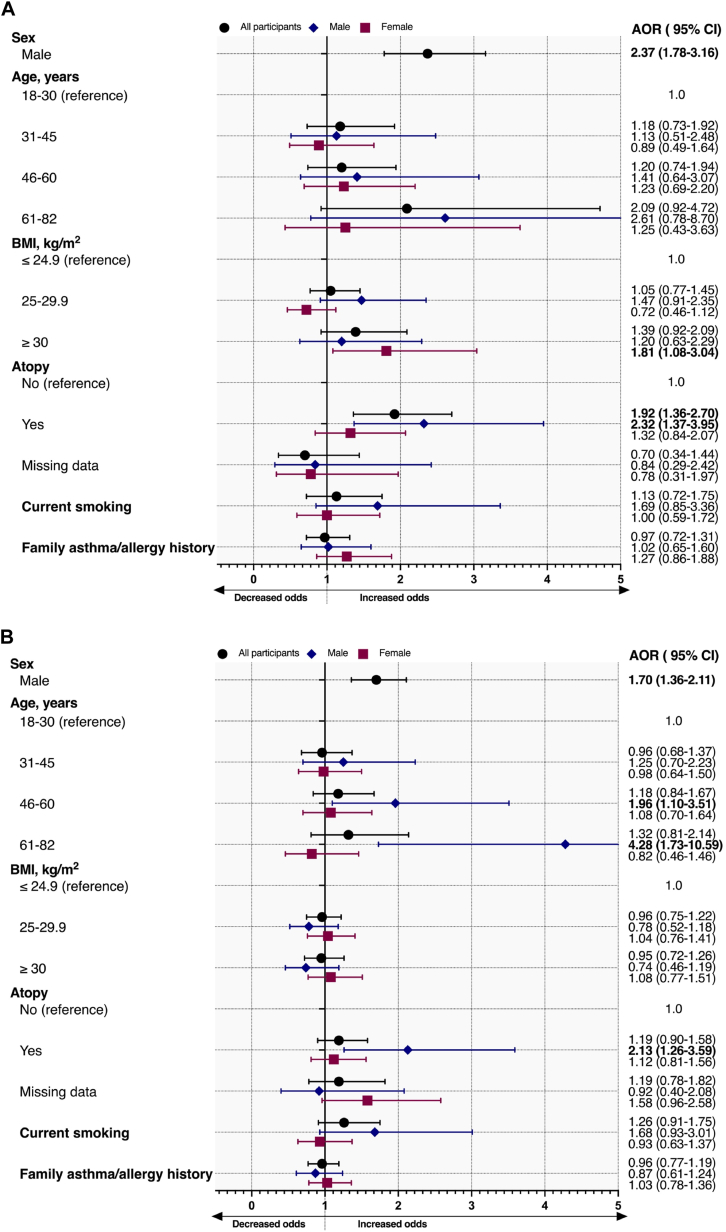
**A** and **B,** Determinants of high serum EDN levels (>75th percentile) stratified by sex in the random adult population (Fig 1, *A*) and subjects with current asthma (Fig 1, *B*). Results were adjusted for sex, age, current smoking, BMI, family history of asthma/allergy, and atopy. Sex-specific models were adjusted for age, current smoking, BMI, family history of asthma/allergy, and atopy. Whiskers demonstrate upper and lower limits of CIs. *BMI*, Body mass index.

1. *Random adult population:* Male sex was associated with increased odds of high serum (adjusted odds ratio [AOR], 2.37; 95% CI, 1.78-3.16) and NLF (AOR, 1.89; 95% CI, 1.24-2.88) EDN levels (Figs 1, *A*, and 2, *A*). Current smoking was not related to high serum and NLF EDN. Obesity was associated with increased odds of high serum EDN levels in female participants (AOR, 1.81; 95% CI, 1.08-3.04), but not in male participants (AOR, 1.20; 95% CI, 0.63-2.29) (Fig 1, *A*). Lastly, subjects with atopy had higher odds of high serum (AOR, 1.92; 95% CI, 1.36-2.70) and NLF (AOR, 1.98; 95% CI, 1.22-3.20) EDN levels. The same findings were observed in male participants, but less so in female participants (Figs 1, *A*, and 2, *A*).

**Fig 2:**
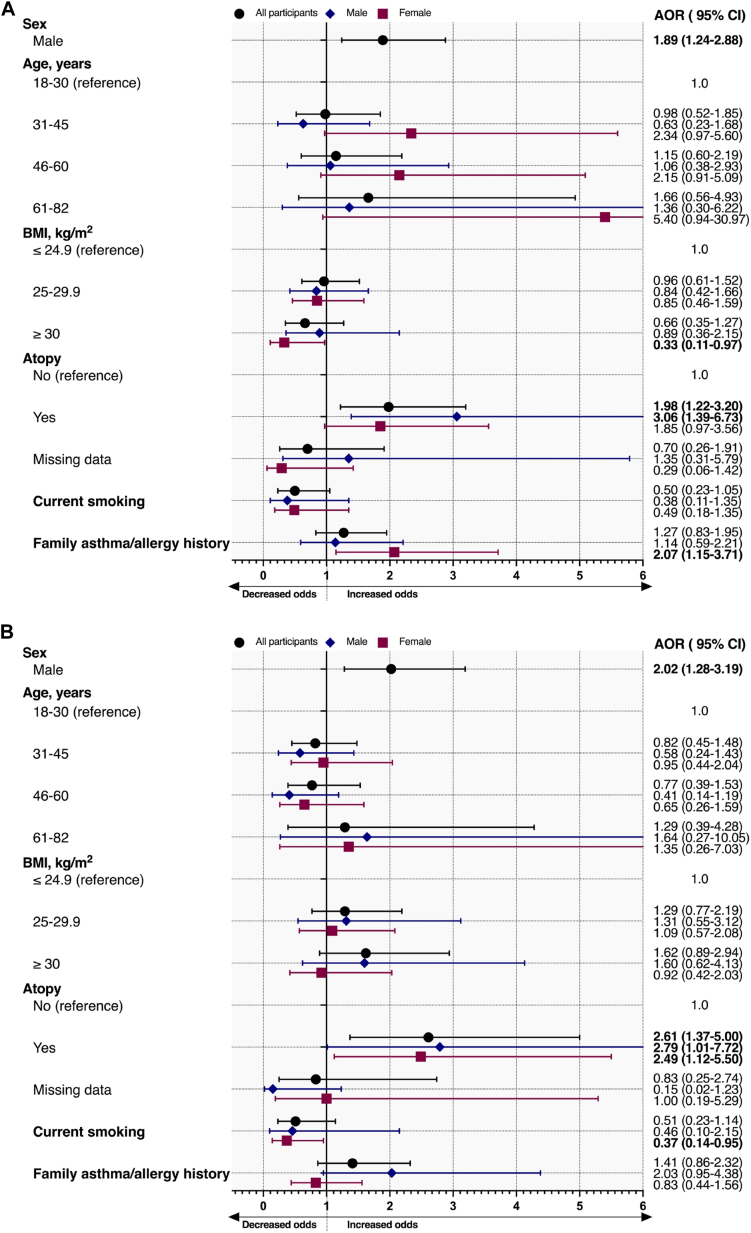
**A** and **B,** Determinants of high NLF EDN levels (>75th percentile) stratified by sex in the random adult population (Fig 2, *A*) and subjects with current asthma (Fig 2, *B*). Results were adjusted for sex, age, current smoking, BMI, family history of asthma/allergy, and atopy. Sex-specific models were adjusted for age, current smoking, BMI, family history of asthma/allergy, and atopy. Whiskers demonstrate upper and lower limits of CIs. *BMI*, Body mass index.
2. *Subjects with current asthma:* Male subjects with current asthma were more likely to have high serum and NLF EDN levels than female subjects with current asthma (Figs 1, *B*, and 2, *B*). Obesity was not associated with serum or NLF EDN levels. Atopy was associated with increased odds of high serum EDN levels in male subjects (AOR, 2.13; 95% CI, 1.26-3.59), but not in female subjects (AOR, 1.12; 95% CI, 0.81-1.56) (Fig 1, *B*). However, atopy was associated with increased odds of high NLF EDN in both male (AOR, 2.79; 95% CI, 1.01-7.72) and female (AOR, 2.49; 95% CI, 1.12-5.50) subjects in those with current asthma (Fig 2, *B*).
3. *Subjects without asthma, any allergic disease, or atopy:* Male subjects without asthma, any allergic disease, or atopy were more likely to have high serum EDN levels (AOR, 1.97; 95% CI, 1.28-3.04) than female subjects, but not high NLF EDN levels (AOR, 1.44; 95% CI, 0.65-3.16) (see Figs E10 and E11 in this article’s Online Repository at www.jaci-global.org). Obesity was related to increased odds of high serum EDN levels, whereas being overweight was not. Female subjects who had obesity were more likely to have high serum EDN levels, but this was not the case in male subjects (Fig E10).
4. *All study participants:* Male sex and atopy were associated with high serum and NLF EDN levels in all participants. Determinants of high EDN levels for all study participants are shown in Figs E12 and E13.

### Clinical determinants of high EDN levels

**Fig 3:**
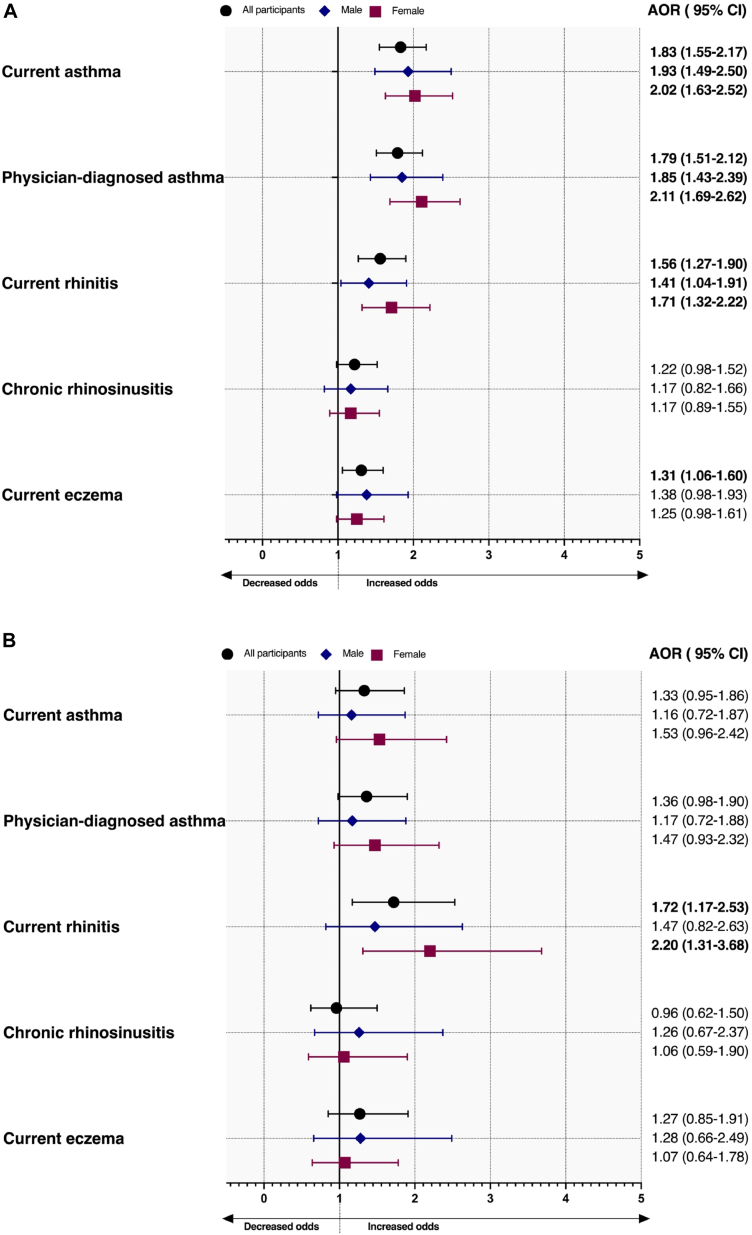
**A** and **B,** Current asthma, physician-diagnosed asthma ever, current rhinitis, chronic rhinosinusitis, and current eczema risk in relation to high serum EDN levels (>75th percentile) (Fig 3, *A*) and high serum NLF EDN levels (>75th percentile) (Fig 3, *B*) in all participants. Whiskers demonstrate upper and lower CIs. Results were adjusted for sex, age, current smoking, BMI, family history of asthma/allergy, and atopy. Sex-specific models were adjusted for age, current smoking, BMI, family history of asthma/allergy, and atopy. *BMI*, Body mass index.

Current asthma was associated with increased odds of high serum EDN levels in all participants (AOR, 1.83; 95% CI, 1.55-2.17), male participants (AOR, 1.93; 95% CI, 1.49-2.50), and female participants (AOR, 2.02; 95% CI, 1.63-2.52) (Fig 3, *A*). Similarly, physician-diagnosed asthma ever (AOR, 1.79; 95% CI, 1.51-2.12), current rhinitis (AOR, 1.56; 95% CI, 1.27-1.90), and current eczema (AOR, 1.31; 95% CI, 1.06-1.60) were also significantly associated with increased odds of high serum EDN levels. Current eczema did not remain significant in sex-stratified models. There was no significant relationship between high serum EDN levels and chronic rhinosinusitis in all models (Fig 3, *A*).

For NLF EDN, current asthma and physician-diagnosed asthma ever were not associated with high levels (Fig 3, *B*). Subjects with current rhinitis were more likely to have high NLF EDN levels. However, this was observed only in female subjects. Chronic rhinosinusitis did not demonstrate any significant association with high NLF EDN levels. Lastly, there was no significant relationship between current eczema and high NLF EDN levels (Fig 3, *B*).

### Correlation with peripheral blood eosinophil count and Feno levels

**Fig 4:**
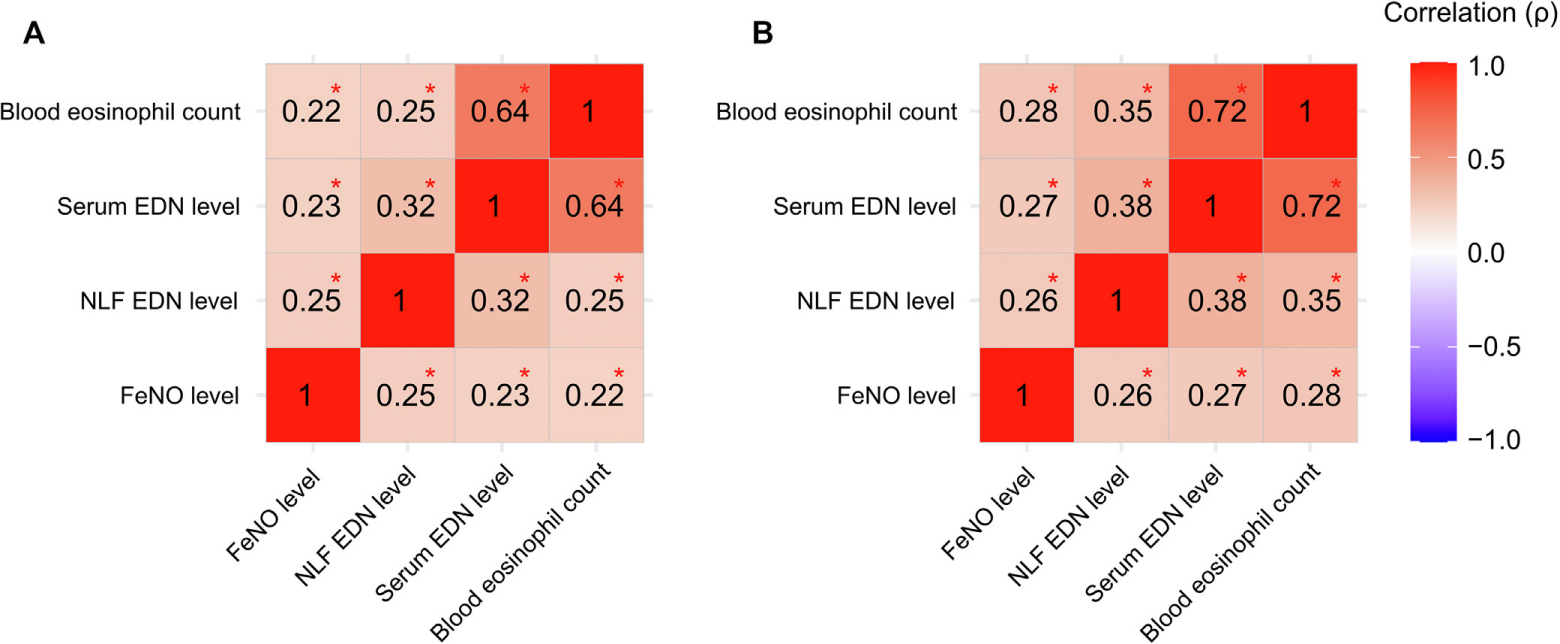
**A** and **B,** Correlation matrix (Spearman ρ) between serum EDN levels, NLF EDN levels, blood eosinophil count, and Feno levels in the random adult population (Fig 4, *A*) and in those with current asthma (Fig 4, *B*). Significant correlations are marked with asterisks.

Serum and NLF EDN levels demonstrated a weak positive correlation in the random adult population (*r*_s_ = 0.32; *P* < .001) (Fig 4). There was a moderate correlation between blood eosinophil count and serum EDN levels (*r*_s_ = 0.64; *P* < .001), whereas the correlation between blood eosinophil count and NLF EDN levels was weak *(r*_s_ = 0.25; *P* < .001) in the random adult population. Both serum and NLF EDN levels showed weak correlation with Feno levels. The correlation between serum EDN, NLF EDN, Feno, and blood eosinophil count remained significant in male and female subjects except for Feno and serum EDN levels in male subjects (see Fig E14 in this article’s Online Repository at www.jaci-global.org). The correlation (Spearman ρ) between these biomarkers is shown in Fig E15 (in the Online Repository available at www.jaci-global.org).

## Discussion

### Summary of key findings

In our population-representative adult cohort, we found that both serum and NLF EDN levels showed significant sex differences regardless of the population subgroups—random sample or asthma sample—with male subjects consistently having higher levels than female subjects. Being obese was significantly associated with increased odds of high serum EDN in female subjects in the random adult population. Conversely, atopy was associated with high serum EDN levels in male subjects but not in female subjects in the random adult population. Those with current asthma had increased odds of having high serum EDN levels, but not high NLF EDN levels. High EDN levels were also associated with different demographic and clinical determinants in stratified analyses on the basis of sex and sampling method (ie, serum vs nasal fluid).

### Strengths and limitations

To our knowledge, the present study is the largest and first population-based study that measured EDN levels in both serum and NLF in adults in a population-based setting. Furthermore, we used a study population representative of adults in western Sweden. A strength of the study is that EDN was measured using a standardized automated ImmunoCAP assay with documented high analytical performance.^25^ However, it should be noted that cross-sectional design of the present study does not infer a direct causality and longitudinal studies are warranted.

Approximately 17% of the NLF samples yielded results below the lower limit of the calibration curve of the EDN assay, leading to left-censored data, but this was handled by a Gibbs sampler imputation method.^32^ Although left-censored data might be associated with the analytical performance of the assay, unknown dilution of the NLF samples also needs to be considered.^33^, ^34^, ^35^ Although the same sampling procedure has been used throughout the present study, dilution during the sample collection may show variation related to absorption or lavage fluid being swallowed.^33^ In addition, mediator levels depend on the sample collection technique.^33^ Accordingly, in addition to imputation of the left-censored data, methodological differences and unknown dilution of the nasal samples should be taken into account to interpret the findings of the present study. In addition, EDN might show time and temperature dependency, and hence variations in blood sampling could also affect the results.^36^ Therefore, standardization of sample collection for both serum and NLF samples might improve interpretation and diagnostic accuracy. In addition, different assay methods have been adopted to measure EDN levels including radioimmunoassay and ELISA in several samples (serum, urine, and sputum) in studies of different populations, which increases the complexity of the interpretation of the results.^13^, ^14^, ^15^^,^^37^

Anti–IL-5 therapies could also affect EDN levels. The use of biologics was nonexistent in our study; therefore, we did not adjust for their use. Longitudinal studies evaluating the effect of anti–IL-5 therapies on EDN levels, particularly as a prognostic marker, are needed to overcome this limitation. Lastly, other primary or secondary eosinophilic disorders, such as eosinophilic esophagitis, allergic bronchopulmonary aspergillosis, chronic eosinophilic pneumonia, and parasitic infections, were not available in our data but might affect EDN levels^38^; these associations need further investigation.

Despite the aforementioned limitations, the present study provides a detailed description of serum and NLF EDN levels and their determinants in different subpopulations by using an automatized method. Considering that there is lack of well-established EDN reference values for both serum and NLF samples, the present work provides detailed information about the distribution of EDN levels in the random adult population.

### Comparison with previous findings

Previous studies have showed that males have higher blood eosinophil count and Feno levels than do females^28^^,^^39^, ^40^, ^41^; however, the same threshold values have still been applied for both sexes in the clinical and research settings. Similarly, a recent study suggests that age and sex should be taken into account in interpreting blood eosinophil count levels.^40^ Our data suggest that EDN levels also differ between males and females, with males having higher EDN levels. In line with our findings, a recent pediatric population–based study demonstrated that 1- to 3-year-old boys had higher serum EDN results than did girls.^23^ However, in contrast to our findings, this difference was not observed in those without atopy and other subpopulations.^23^ Hence, the authors argued that the sex-related differences could be driven by the presence of atopy, which is more frequent in boys than in girls, particularly during the prepubertal era.^23^ Similar to our findings, a recent study reported this sex difference in serum EDN in a healthy population aged 50 to 64 years.^24^ Our data go beyond these previous findings by demonstrating that serum and NLF EDN levels are higher in males than in females during adulthood.

The observed sex differences in EDN levels also have a key clinical implication. Importantly, the application of the same thresholds for EDN levels without considering sex could be misleading in the clinical setting. Using sex-specific thresholds could provide a more accurate interpretation on an individual basis, and clinical implications with these differences in mind should be investigated in future studies. Moreover, the underlying mechanisms for sex differences remain unclear for environmental exposures, genetic/hormonal factors, and concomitant diseases.

Regardless of sex, subjects with current asthma in the present study were more likely to have high serum EDN levels than those without. The findings for NLF EDN measurements attenuated to nonstatistical significance in adjusted models. To date, a limited number of studies have investigated the NLF EDN levels in relation to asthma and wheezing and, to our knowledge, all previous studies we know about have been conducted in children.^35^^,^^42^, ^43^, ^44^

A previous study found that NLF EDN levels were higher in children with asthma than in those without.^44^ In the present study, subjects with asthma and rhinitis had higher EDN levels than those without, both in serum and in NLF. However, in adjusted models, only serum EDN levels, but not NLF EDN levels, differed regarding the presence of current asthma and current eczema. This may indicate that NLF samples could reflect local inflammation as being more specific than serum samples, whereas serum samples reflect systemic inflammation. In addition, there was a weak positive correlation between serum and NLF EDN levels in our findings. Although there is conflicting data whether nasal samples could reflect bronchial epithelium,^45^, ^46^, ^47^ serum and nasal EDN levels might reflect different activation of nasal and systemic inflammation.

Interestingly, high serum and NLF EDN levels did not show any differences in those with chronic rhinosinusitis compared with those without. In support, a recent study did not reveal any association between serum EDN levels and chronic sinusitis.^24^ Because chronic rhinosinusitis is defined on the basis of the epidemiologic definition in our study, further investigation with clinical definition is needed to confirm present findings.

In addition, allergen exposure could affect NLF EDN levels. In a previous study, NLF EDN levels increased in late response to nasal antigen challenge, peaking at 8 hours after exposure.^48^ Therefore, seasonal exposure of pollen and molds might influence EDN levels in sensitized subjects and needs further investigation. Nevertheless, NLF EDN has several advantages such as being less invasive, easy to perform, and able to reflect local inflammation. Future studies investigating NLF EDN levels and disease outcomes are needed, especially in adults.

Interestingly, obesity was associated with high serum EDN levels in females, but not in males. In line with our findings, obesity has been associated with increased blood eosinophil levels.^28^^,^^40^ Nevertheless, the relationship between adiposity and eosinophilic activation has not been fully elucidated.^49^ Although some studies suggest that eosinophils have a protective effect on obesity and play a role in adipose tissue homeostasis,^50^^,^^51^ contradictory results have also been revealed and neither relationship nor mechanism is so far fully understood.^49^ We did not find any positive association between obesity and NLF EDN. It might be argued that serum EDN levels could be related to systemic low-grade inflammation in subjects with obesity,^52^ whereas NLF EDN levels might not reflect this aspect.

### Conclusion

This study provides a comprehensive overview of serum and NLF EDN levels and their determinants in different subpopulations in western Sweden, including a representative random adult population, a current asthma sample, and a population without asthma, any allergic disease, or atopy. Both serum and NLF EDN levels demonstrate significant sex-related differences, with males consistently having higher values than females. Those with current asthma were more likely to have higher serum, but not NLF, EDN levels. Although serum EDN appears as a promising biomarker in asthma, associated factors—particularly sex, obesity, and atopy—should be taken into account in interpreting EDN levels.

**Clinical implications**

EDN levels are influenced by subjects’ characteristics—particularly sex, obesity, and atopy—highlighting the need to consider them when interpreting EDN values in clinical practice.

## Disclosure statement

This study was funded by the VBG Group Herman Krefting Foundation for Asthma and Allergy Research, Sweden; the 10.13039/501100010234Swedish Asthma and Allergy Association, ALF agreement (grants from the Swedish state under the agreement between the Swedish government and the county councils, Västra Götaland); the Swedish Heart-Lung Foundation; and the 10.13039/501100004359Swedish Research Council. Research kits were provided by Thermo Fisher Scientific (Uppsala, Sweden) on behalf of the West Sweden Asthma Study.

Disclosure of potential conflict of interest: S. S. Ö. Ermis reports conference attendance–related fees from 10.13039/100011033Thermo Fisher Scientific. M. P. Borres and R. Movérare are employees of Thermo Fisher Scientific. H. Kankaanranta reports fees for consultancies and lectures from AstraZeneca, Boehringer Ingelheim, Chiesi Pharma, Covis Pharma, GlaxoSmithKline, MSD, Orion Pharma, and Sanofi, outside the submitted work. R. Abohalaka reports travel grants for international meetings from the 10.13039/100001465American Thoracic Society, the 10.13039/100008593European Respiratory Society, the 10.13039/501100003793Swedish Heart-Lung Foundation, and the Adlerbertska Foundation, outside the submitted work. The rest of the authors declare that they have no relevant conflicts of interest.
