# Supplementary material for: Serum and nasal lavage fluid eosinophil-derived neurotoxin levels and their determinants in adults
Source: J Allergy Clin Immunol Glob. 2025 Jun 6;4(3):100510. doi: 10.1016/j.jacig.2025.100510 (PMC12270069; doi:10.1016/j.jacig.2025.100510)
Supplement: Supplementary Figs and Tables [file mmc1.docx]

**ONLINE REPOSITORY**

**Serum and nasal lavage fluid eosinophil-derived neurotoxin levels and their determinants in adults**

Saliha Selin Özuygur Ermis^1^, Carina Malmhäll^1^, Magnus P. Borres^2,3^, Robert Movérare^3,4^, Daniil Lisik^1^, Reshed Abohalaka^1^, Selin Ercan^1^, Susanne Schmeisser^5^, Rani Basna^1,6^, Roxana Mincheva^1^, Göran Wennergren^7^, Jan Lötvall^1^ , Linda Ekerljung^1,8^, Madeleine Rådinger^1^, Hannu Kankaanranta^1,9,10^, Bright I. Nwaru^1,11^

^1^Krefting Research Centre, Institute of Medicine, Sahlgrenska Academy, University of Gothenburg, Gothenburg, Sweden

^2^Department of Women’s and Children’s Health, Uppsala University, Uppsala, Sweden

^3^Thermo Fisher Scientific, Uppsala, Sweden

^4^Department of Medical Sciences: Respiratory, Allergy and Sleep Research, Uppsala University, Uppsala, Sweden.

^5^Department of Clinical Immunology, Sahlgrenska University Hospital, Gothenburg, Sweden

^6^Department of Clinical Science, Lund University, Lund, Sweden

^7^Department of Paediatrics, Sahlgrenska Academy, University of Gothenburg, Gothenburg, Sweden

^8^Department of Respiratory Medicine and Allergology, Sahlgrenska University Hospital, Gothenburg, Sweden

^9^Tampere University Respiratory Research Group, Faculty of Medicine and Health Technology, Tampere University, Tampere, Finland

^10^Department of Respiratory Medicine, Seinäjoki Central Hospital, Seinäjoki, Finland

^11^Wallenberg Centre for Molecular and Translational Medicine, University of Gothenburg, Gothenburg, Sweden

Corresponding author

Saliha Selin Özuygur Ermis

Krefting Research Centre, Institute of Medicine, Sahlgrenska Academy

University of Gothenburg, Gothenburg, Sweden

Medicinaregatan 1F, Box 424, 405 30 Gothenburg, Sweden

[selin.ozuygur@gu.se](mailto:selin.ozuygur@gu.se)

**ORCID list of the authors:**

| Saliha Selin Özuygur Ermis | 0000-0003-3507-773X |
| --- | --- |
| Carina Malmhäll | 0000-0001-6696-7570 |
| Rani Basna | 0000-0001-7510-8460 |
| Magnus P. Borres | 0000-0002-9045-2304 |
| Robert Movérare | 0000-0001-6611-5036 |
| Daniil Lisik | 0000-0002-0220-5961 |
| Reshed Abohalaka | 0000-0003-2803-2912 |
| Selin Ercan | 0000-0002-9356-3042 |
| Roxana Mincheva | 0000-0002-6072-2748 |
| Susanne Schmeisser | N/A |
| Jan Lötvall | 0000-0001-9195-9249 |
| Emma Goksör | 0000-0001-9595-1877 |
| Linda Ekerljung | 0000-0001-5784-0041 |
| Göran Wennergren | 0000-0002-7010-7191 |
| Madeleine Rådinger | 0000-0002-0652-7378 |
| Hannu Kankaanranta | 0000-0001-5258-0906 |
| Bright Ibeabughichi Nwaru | 0000-0002-2876-6089 |

**ONLINE REPOSITORY INDEX**

1. **Supplementary Material and Method**
   1. **Whole blood collection for cell count and serum samples in WSAS**
   2. **Collection of nasal lavage fluid samples in WSAS**
   3. **Imputation of missing data for fractional exhaled nitric oxide (FE_NO_) and blood eosinophil count**
2. **Supplementary Tables**

**Table E1.** Baseline characteristics of subjects either with or without nasal lavage fluid EDN levels in random adult population.

**Table E2.** Serum and nasal lavage fluid EDN levels regarding the presence of current asthma, physician-diagnosed asthma ever, current rhinitis, current allergic rhinitis, chronic rhinosinusitis, and presence of asthma, any allergic condition or atopy in random adult population.

**Table E3.** Serum and nasal lavage fluid EDN levels stratified by sex categories regarding the presence of current asthma, physician-diagnosed asthma ever, current rhinitis, current allergic rhinitis, chronic rhinosinusitis, and presence of asthma, any allergic condition or atopy in random adult population.

**Table E4.** Blood eosinophil count and FeNO levels in relation to sex in different study subpopulations.

1. **Supplementary Figures**

**Figure E1.** Flow chart of the study participants with serum EDN levels (n = 2,939). **Details.** *Since 22 cases participated more than one visit, latest visit was excluded in case of multiple measurements. **Abbreviations.** EDN= Eosinophil-derived neurotoxin.

**Figure E2.** Flow chart of the study participants with nasal lavage fluid EDN levels (n = 878). **Details.** *Subjects without NLF cell count was also not included. **Abbreviations.** EDN= Eosinophil-derived neurotoxin, NLF= Nasal lavage fluid.

**Figure E3.** Violin plot of serum and nasal lavage fluid EDN levels in random adult population stratified by sex (truncated). **Abbreviations.** EDN= Eosinophil-derived neurotoxin, NLF=Nasal lavage fluid, Q= quartile.

**Figure E4.** Receiver operating characteristic curve for serum EDN level (µg/l) in current asthma vs without current asthma **(Figure E4A)** and current rhinitis vs without current rhinitis **(Figure E4B**). **Details.** Threshold values calculated based on Youden index. **Abbreviations.** AUC= Area under curve, EDN= Eosinophil-derived neurotoxin.

**Figure E5.** Receiver operating characteristic curve for nasal lavage fluid EDN level (µg/l) in current asthma vs without current asthma **(Figure E5A)** and current rhinitis vs without current rhinitis **(Figure E5B).** **Details.** Threshold values calculated based on Youden index. **Abbreviations.** AUC= Area under curve, EDN= Eosinophil-derived neurotoxin.

**Figure E6.** Receiver operating characteristic curve for serum EDN level (µg/l) in current asthma vs without current asthma **(Figure E6A)** and current rhinitis vs without current rhinitis in males **(Figure E6B**). **Details.** Threshold values calculated based on Youden index. **Abbreviations.** AUC= Area under curve, EDN= Eosinophil-derived neurotoxin.

**Figure E7.** Receiver operating characteristic curve for nasal lavage fluid EDN level (µg/l) in current asthma vs without current asthma **(Figure E7A)** and current rhinitis vs without current rhinitis in males **(Figure E7B).** **Details.** Threshold values calculated based on Youden index. **Abbreviations.** AUC= Area under curve, EDN= Eosinophil-derived neurotoxin.

**Figure E8.** Receiver operating characteristic curve for serum EDN level (µg/l) in current asthma vs without current asthma **(Figure E8A)** and current rhinitis vs without current rhinitis in females **(Figure E8B**). **Details.** Threshold values calculated based on Youden index. **Abbreviations.** AUC= Area under curve, EDN= Eosinophil-derived neurotoxin.

**Figure E9.** Receiver operating characteristic curve for nasal lavage fluid EDN level (µg/l) in current asthma vs without current asthma **(Figure E9A)** and current rhinitis vs without current rhinitis in females **(Figure E9B).** **Details.** Threshold values calculated based on Youden index. **Abbreviations.** AUC= Area under curve, EDN= Eosinophil-derived neurotoxin.

**Figure E10.** Determinants of high serum EDN levels (above 75^th^ percentile) stratified by sex in subjects without asthma, any allergic disease, or atopy. **Details.** Results were adjusted for sex, age, current smoking, BMI, family history of asthma/allergy. Sex-specific models were adjusted for age, current smoking, BMI, family history of asthma/allergy. Whiskers demonstrate upper and lower limits of confidence intervals. **Abbreviations.** AOR= Adjusted odds ratio, BMI= Body mass index, CI= Confidence interval.

**Figure E11.** Determinants of high NLF EDN levels (above 75^th^ percentile) stratified by sex in subjects without asthma, any allergic disease, or atopy. **Details.** Results were adjusted for sex, age, current smoking, BMI, family history of asthma/allergy. Sex-specific models were adjusted for age, current smoking, BMI, family history of asthma/allergy. Whiskers demonstrate upper and lower limits of confidence intervals. **Abbreviations.** AOR= Adjusted odds ratio, BMI= Body mass index, CI= Confidence interval.

**Figure E12.** Determinants of high serum EDN levels (above 75^th^ percentile) stratified by sex in all participants. **Details.** Results were adjusted for sex, age, current smoking, BMI, family history of asthma/allergy, and atopy. Sex-specific models were adjusted for age, current smoking, BMI, family history of asthma/allergy, and atopy. Whiskers demonstrate upper and lower limits of confidence intervals. **Abbreviations.** AOR= Adjusted odds ratio, BMI= Body mass index, CI= Confidence interval.

**Figure E13**. Determinants of high NLF EDN levels (above 75^th^ percentile) stratified by sex in all participants. **Details.** Results were adjusted for sex, age, current smoking, BMI, family history of asthma/allergy, and atopy. Sex-specific models were adjusted for age, current smoking, BMI, family history of asthma/allergy, and atopy. Whiskers demonstrate upper and lower limits of confidence intervals. **Abbreviations.** AOR= Adjusted odds ratio, BMI= Body mass index, CI= Confidence interval.

**Figure E14**. Correlation matrix (Spearman’s rho) between serum EDN levels, NLF EDN levels, blood eosinophil count, and FeNO levels stratified in males in random adult population and in those with current asthma. **Details.** Significant correlations were marked with asterisks. Abbreviations. EDN= Eosinophil-derived neurotoxin, FeNO= Fractional exhaled nitric oxide, NLF = Nasal lavage fluid.

**Figure E15.** Correlation (Spearman’s rho) in the random adult population between serum EDN level (µg/l) and blood eosinophil count **(A)**; serum EDN level and FeNO level (ppb) **(B)**; NLF EDN level (µg/l) and blood eosinophil count **(C)**; NLF EDN levels and FeNO levels **(D)**; correlation between serum EDN levels and NLF EDN levels **(E).** **Abbreviations.** EDN= Eosinophil-derived neurotoxin, FeNO= Fractional exhaled nitric oxide, NLF = Nasal lavage fluid.

1. **Supplementary Material and Method**
   1. **Whole blood collection for cell count and serum samples in WSAS**

Due to contraindications, the procedure was not performed in those with bilateral mastectomy history, edema/hematoma/scars in the area, or those who did not give consent. The procedure was not performed on the same side as the mastectomy.

Following a 15-minute rest period, the arm of the participant was placed in a flat or slightly sloping downward position. A band was placed 7-10 cm above the antecubital fossa. After disinfection of skin, 30 seconds were given to dry. During the venipuncture procedure, with a maximum 30-degree angle, the cannula opening face was inserted in an upward position. In the serum tube, approximately 14 ml venous blood is collected. After sampling, arm was released, and tubes were rotated back to forth at least 5 times. Venous blood samples for serum were collected in SST tubes containing separating gel and clot activator. Samples were allowed to coagulate at room temperature for 60 minutes before centrifugation. If blood has not coagulated properly, samples might be allowed for another 30 minutes before centrifugation. After 60 min at room temperature, tubes were centrifuged for 10 min at 1880xg, +8 degree (Heraeus Multifuge ® 1 S-R, Kendro Laboratory Products, Langenselbold, Germany). Serum samples were aliquoted and stored at -80°C until analysis. Samples that were not subjected to freeze-thaw cycles were used for the analysis.

Whole blood for differential cell counts were collected in K2EDTA tubes and measured at Clinical chemistry Laboratory, Sahlgrenska University Hospital, Gothenburg, Sweden.

- 1. **Collection of nasal lavage fluid samples in WSAS**

The procedure was not performed on subjects with nostril congestion and absence of nasal septum.

To provide pharynx closure, study participants were requested to be positioned with their heads slightly tilted back at 30 degrees. Then, a 0.9% sterile saline solution (5 ml, at room temperature) was given into the nostrils. During the procedure, participants were requested to lock their pharynx to prevent the escape to the throat. After this step, study participants requested to bend their heads forward and keep their mouths closed at the same time, then run out the nasal rinse liquid into the bowl in front of them.

Collected nasal rinse fluid was transferred into a 15 ml tube on ice. The collected sample was centrifuged at 300xg at 4°C for 10 minutes. The NLF supernatant was aliquoted and stored at -80°C  until analysis. The remaining cell pellet was resuspended in PBS. Cytospins were prepared and stained with May Grunwald- and Giemsa stain (HistoLab Products AB, Gothenburg, Sweden) for differential cell count using a Zeiss Axioplan microscope (Carl Zeiss Jena GmbH, Eching, Germany).

- 1. **Imputation of missing data for fractional exhaled nitric oxide (FE_NO_) and blood eosinophil count**

Of 878, 1% had missing blood eosinophil count and 9% had missing FE_NO_ data. FE_NO_ and blood eosinophil levels were imputed using multiple imputation by chained equations (MICE) with random forests (MICE-RF), implemented with the miceRanger R package. MICE-RF has been shown to produce reliable estimates.^1^ The model converged well within 20 iterations, and 100 imputed datasets were generated to account for uncertainty in the imputation.^2^ Mean of 100 imputed values were calculated and used for data analysis.

**References**

E1. Shah AD, Bartlett JW, Carpenter J, Nicholas O, Hemingway H. Comparison of random forest and parametric imputation models for imputing missing data using MICE: a CALIBER study. Am J Epidemiol. Mar 15 2014;179(6):764-74. doi:10.1093/aje/kwt312

E2. White IR, Royston P, Wood AM. Multiple imputation using chained equations: Issues and guidance for practice. Statistics in Medicine. 2011/02/20 2011;30(4):377-399. doi:<https://doi.org/10.1002/sim.4067>

1. **Supplementary Tables**

**Table E1.** Baseline characteristics of subjects either with or without nasal lavage fluid EDN levels in random adult population.

|  | Missing data in random adult population  (Not measured for NLF EDN)  *N=* 645 | Non-missing data in random adult population (Measured for NLF EDN)  *n =* 527 | *p*-value |
| --- | --- | --- | --- |
| Age, years in strata, n (%) |  |  | **0.002** |
| 18-30 | 72 (44.2) | 91 (55.8) |  |
| 31-45 | 147 (50.9) | 142 (49.1) |  |
| 46-60 | 211 (58.8) | 148 (41.2) |  |
| 61-82 | 215 (59.6) | 146 (40.4) |  |
| Sex, n (%) |  |  | 0.788 |
| Male, | 296 (54.6) | 246 (45.4) |  |
| Female, | 349 (55.4) | 281 (44.6) |  |
| Body mass index, kg/m^2^ ,n (%) |  |  | 0.050 |
| ≤24.9 | 249 (50.9) | 240 (49.1) |  |
| 25-29.9 | 294 (58.6) | 208 (41.4) |  |
| ≥30 | 102 (56.4) | 79 (43.6) |  |
| Smoking status, n (%) |  |  |  |
| Ever smoking |  |  | 0.175 |
| No | 339 (57.0) | 256 (43.0) |  |
| Yes | 306 (53.0) | 271 (47.0) |  |
| Current smoking |  |  | 0.602 |
| No | 573 (55.3) | 463 (44.7) |  |
| Yes | 72 (52.9) | 64 (47.1) |  |
| Family asthma/allergy history,  n (%) |  |  | 0.329 |
| No | 412 (56.1) | 322 (43.9) |  |
| Yes | 233 (53.2) | 205 (46.8) |  |
| Atopy, n (%) |  |  | **0.005** |
| No | 257 (54.3) | 216 (45.7) |  |
| Yes | 163 (49.1) | 169 (50.9) |  |
| Missing data/non-valid results | 225 (61.3) | 142 (38.7) |  |
| Current asthma, n (%) |  |  | 0.131 |
| No | 562 (56.0) | 442 (44.0) |  |
| Yes | 83 (49.7) | 84 (50.3) |  |
| Physician-diagnosed asthma ever, n (%) |  |  | 0.100 |
| No | 576 (55.9) | 454 (44.1) |  |
| Yes | 69 (48.6) | 73 (51.4) |  |
| Current rhinitis, n (%) |  |  | 0.064 |
| No | 312 (57.9) | 227 (42.1) |  |
| Yes | 329 (52.5) | 298 (47.5) |  |
| Current allergic rhinitis, n (%) |  |  | **0.020** |
| No | 407 (56.3) | 316 (43.7) |  |
| Yes | 122 (47.8) | 133 (52.2) |  |
| Chronic rhinosinusitis, n (%) |  |  | 0.643 |
| No | 584 (55.3) | 473 (44.7) |  |
| Yes | 55 (52.9) | 49 (47.1) |  |
| Current eczema, n (%) |  |  | 0.367 |
| No | 544 (55.2) | 441 (44.8) |  |
| Yes | 78 (51.3) | 74 (48.7) |  |
| Presence of asthma, any allergic disease or atopy, n (%) |  |  | 0.166 |
| No | 203 (54.9) | 167 (45.1) |  |
| Yes | 246 (50.1) | 245 (49.9) |  |

**Details.** Percentages calculated based on rows. **Abbreviations.** EDN= eosinophil-derived neurotoxin, NLF= Nasal lavage fluid.

**Table E2.** Serum and nasal lavage fluid EDN levels regarding the presence of current asthma, physician-diagnosed asthma ever, current rhinitis, current allergic rhinitis, chronic rhinosinusitis, and presence of asthma, any allergic condition or atopy in random adult population.

|  | Serum EDN levels (µg/l) | | | | | NLF EDN levels (µg/l) | | | | |
| --- | --- | --- | --- | --- | --- | --- | --- | --- | --- | --- |
| Random adult population | ***N*** | **Median (Q1, Q3)** | **P5-95** | **Min-max** | | ***N*** | **Median (Q1, Q3)** | **P5-95** | **Min-max** | |
| All participants | 1142 | 28.9 (20.4-41.9) | 12.6-71.6 | 1.0-346.5 | | 527 | 6.6 (2.8-14.2) | 0-54.4 | 0-243 | |
| Current asthma | ***N*** | **Median (Q1, Q3)** | **P5-95** | **Min-max** | ***p-*value** | ***N*** | **Median (Q1, Q3)** | **P5-95** | **Min-max** | ***p-*value** |
| No | 978 | 28.1 (19.8-40.5) | 12.7-69.3 | 1.0-346.5 | **<0.001** | 442 | 6.4 (2.8-14.0) | 0-48.5 | 0-243.0 | 0.150 |
| Yes | 163 | 32.0 (23.1-50.8) | 12.2-93.1 | 5.6-122.0 |  | 84 | 7.6 (2.9-19.4) | 0-123.8 | 0-196.0 |  |
| Physician-diagnosed asthma ever |  |  |  |  |  |  |  |  |  |  |
| No | 1003 | 28.1 (19.9-40.6) | 12.7-70.8 | 1.0-346.5 | **0.004** | 454 | 6.3 (2.7-13.7) | 0.48.8 | 0-243.0 | **0.035** |
| Yes | 139 | 32.4 (22.4-49.4) | 12.1-97.1 | 5.6-122.0 |  | 73 | 9.4 (3.0-19.3) | 0-142.1 | 0-196.0 |  |
| Current rhinitis |  |  |  |  |  |  |  |  |  |  |
| No | 525 | 28.2 (19.7-39.5) | 13.1-68.1 | 1.0-346.5 | 0.153 | 227 | 5.7 (2.9-10.8) | 0-31.3 | 0-243.0 | **0.003** |
| Yes | 611 | 29.6 (21.0-43.2) | 11.7-73.9 | 5.6-161.0 |  | 298 | 8.0 (2.8-17.6) | 0-62.1 | 0-196.0 |  |
| Current allergic rhinitis |  |  | | | |  |  |  |  |  |
| No | 705 | 27.8 (19.7-39.4) | 12.5-67.6 | 1.0-346.5 | **<0.001** | 316 | 5.6 (2.3-11.6) | 0-39.7 | 0-243.0 | **<0.001** |
| Yes | 246 | 33.0 (23.9-48.3) | 12.2-81.1 | 5.6-133.0 |  | 133 | 10.5 (5.5-26.5) | 0-74.2 | 0-196.0 |  |
| Chronic rhinosinusitis |  |  |  |  |  |  |  |  |  |  |
| No | 1030 | 28.5 (20.2-41.8) | 12.7-71.4 | 1.0-346.5 | 0.159 | 473 | 6.5 (2.8-14.3) | 0-55.4 | 0-243.0 | 0.654 |
| Yes | 101 | 30.5 (22.9-43.3) | 10.8-89.6 | 5.7-136.0 |  | 49 | 7.6 (2.9-13.6) | 0-65.3 | 0-137.0 |  |
| Current eczema |  |  | | | |  |  |  |  |  |
| No | 959 | 27.9 (19.8-41.2) | 12.5-71.2 | 1.0-346.5 | **0.017** | 441 | 6.5 (2.9-14.1) | 0-52.8 | 0-196.0 | 0.516 |
| Yes | 148 | 31.4 (23.6-44.8) | 13.3-84.7 | 7.6-133.0 |  | 74 | 7.1 (2.6-18.5) | 0-71.4 | 0-243.0 |  |
| Atopy |  |  |  |  |  |  |  |  |  |  |
| No | 462 | 26.8 (19.0-37.5) | 11.7-66.8 | 1.0-136.0 | **<0.001** | 216 | 5.0 (2.0-11.3) | 0-41.2 | 0-95.4 | **<0.001** |
| Yes | 319 | 32.0 (23.1-46.4) | 12.7-80.0 | 5.6-346.5 |  | 169 | 9.8 (4.7-23.4) | 0-69.0 | 0-196 |  |
| Presence of asthma, any allergic disease or atopy |  |  |  |  |  |  |  |  |  |  |
| No | 360 | 26.5 (18.5-38.6) | 11.4-67.2 | 1.0-136.0 | **<0.001** | 167 | 5.3 (2.1-12.8) | 0-46.6 | 0-95.4 | **0.001** |
| Yes | 476 | 31.2 (22.6-45.4) | 12.7-80.1 | 5.6-346.5 |  | 245 | 7.9 (3.2-17.4) | 0-66.9 | 0-243.0 |  |

**Details.** Mann-Whitney-U test was performed to compare EDN levels. Presence of asthma, any allergic disease or atopy was defined based on presence of at least one of the following: current asthma, current allergic rhinitis, current eczema, or atopy (defined by at least one skin prick positivity to aeroallergens). **Abbreviations.** EDN= eosinophil-derived neurotoxin, NLF= Nasal lavage fluid, P= percentile, Q= quartile).

**Table E3.** Serum and nasal lavage fluid EDN levels stratified by sex categories regarding the presence of current asthma, physician-diagnosed asthma ever, current rhinitis, current allergic rhinitis, chronic rhinosinusitis, and presence of asthma, any allergic condition or atopy in random adult population.

|  | Serum EDN levels (µg/l) | | | | | NLF EDN levels (µg/l) | | | |  |
| --- | --- | --- | --- | --- | --- | --- | --- | --- | --- | --- |
|  | ***N*** | **Median (Q1, Q3)** | **P5-95** | **Min-max** | ***p*-value** | ***N*** | **Median (Q1, Q3)** | **P5-95** | **Min-max** | ***p*-value** |
| Random adult population |  | | |  |  |  |  | | |  |
| Male | 534 | 34.1 (24.7-46.6) | 15.1-80.0 | 5.6-346.5 | **<0.001** | 246 | 9.0 (4.3-18.3) | 0-60.6 | 0-243.0 | **<0.001** |
| Female | 608 | 24.8 (17.8-34.7) | 11.0-66.5 | 1.0-161.0 |  | 281 | 4.9 (0.8-10.5) | 0-44.3 | 0-158.0 |  |
| Current asthma |  | | |  |  |  |  | | |  |
| Male | 68 | 38.1 (25.7-53.3) | 14.7-97.8 | 5.6-115.0 | **0.010** | 35 | 10.2 (3.2-26.5) | 0-148.8 | 0-196.0 | 0.174 |
| Female | 95 | 29.2 (22.1-45.8) | 10.9-88.9 | 7.6-122.0 |  | 49 | 6.0 (2.6-17.6) | 0-113.5 | 0-158.0 |  |
| Without current asthma |  | | |  |  |  |  | | |  |
| Male | 466 | 33.4 (24.5-45.8) | 15.1-76.3 | 6.5-346.5 | **<0.001** | 211 | 9.0 (4.4-18.1) | 0-60.0 | 0-243.0 | **<0.001** |
| Female | 512 | 23.9 (17.2-33.8) | 11.0-59.7 | 1.0-161.0 |  | 231 | 4.7 (0-10.3) | 0-31.7 | 0-95.4 |  |
| Physician-diagnosed asthma ever |  |  |  |  |  |  |  |  |  |  |
| Male | 65 | 36.0 (25.9-53.3) | 17.5-104.2 | 5.6-115.0 | **0.008** | 30 | 12.5 (5.4-20.2) | 0-163.6 | 0-196 | 0.153 |
| Female | 74 | 29.5 (21.6-43.4) | 10.6-91.0 | 8.6-122.0 |  | 43 | 6.1 (2.8-18.2) | 0-137.2 | 0-158 |  |
| Without physician-diagnosed asthma ever |  |  |  |  |  |  |  |  |  |  |
| Male | 469 | 33.6 (24.4-46.0) | 15.0-77.6 | 6.5-346.5 | **<0.001** | 216 | 8.7 (4.2-18.0) | 0-59.8 | 0-243 | **<0.001** |
| Female | 534 | 24.2 (17.5-34.0) | 11.1-62.5 | 1.0-161.0 |  | 238 | 4.8 (0-10.0) | 0-31.6 | 0-95.4 |  |
| Current rhinitis |  |  |  |  |  |  |  |  |  |  |
| Male | 284 | 34.8 (24.2-47.6) | 13.7-79.7 | 5.6-133.0 | **<0.001** | 146 | 10.3 (4.7-24.0) | 0-61.5 | 0-196 | **<0.001** |
| Female | 327 | 25.5 (18.5-36.3) | 10.6-70.2 | 7.6-161.0 |  | 152 | 6.0 (2.0-13.8) | 0-67.1 | 0-158 |  |
| Without current rhinitis |  |  |  |  |  |  |  |  |  |  |
| Male | 248 | 33.6 (25.0-45.1) | 15.9-79.7 | 11.3-346.5 | **<0.001** | 99 | 7.8 (4.1-14.5) | 0.3-60.7 | 0-243 | **<0.001** |
| Female | 277 | 24.0 (16.7-33.7) | 11.7-61.4 | 1.0-103.0 |  | 128 | 4.1 (0.2-7.4) | 0-25.8 | 0-63.3 |  |
| Current allergic rhinitis |  |  |  |  |  |  |  |  |  |  |
| Male | 127 | 36.0 (27.7-51.0) | 15.3-96.4 | 5.6-133.0 | **<0.001** | 74 | 13.5 (6.3-30.6) | 1.8-71.4 | 0-196 | **0.010** |
| Female | 119 | 27.4 (19.8-42.8) | 10.4-73.8 | 7.6-122.0 |  | 59 | 6.4 (3.3-18.2) | 0-90.0 | 0-158 |  |
| Without current allergic rhinitis |  |  |  |  |  |  |  |  |  |  |
| Male | 309 | 33.6 (25.0-44.5) | 15.3-77.6 | 7.9-346.5 | **<0.001** | 131 | 7.5 (3.6-14.1) | 0-50.9 | 0-243 | **<0.001** |
| Female | 396 | 24.0 (17.0-34.0) | 10.9-59.2 | 1.0-122.0 |  | 185 | 4.2 (0.1-9.3) | 0-29.0 | 0-95.4 |  |
| With chronic rhinosinusitis |  |  |  |  |  |  |  |  |  |  |
| Male | 49 | 35.8 (23.9-48.7) | 17.8-98.7 | 13.5-136.0 | **0.017** | 29 | 9.0 (4.9-22.1) | 0-110.7 | 0-137.0 | **0.034** |
| Female | 52 | 29.3 (20.5-35.3) | 7.9-64.8 | 5.7-67.2 |  | 20 | 4.9 (2.3-9.2) | 0-43.6 | 0-44.7 |  |
| Without chronic rhinosinusitis |  |  |  |  |  |  |  |  |  |  |
| Male | 480 | 33.7 (24.7-46.1) | 14.9-76.3 | 5.6-346.5 | **<0.001** | 215 | 9.0 (4.1-18.1) | 0-60.5 | 0-243.0 | **<0.001** |
| Female | 550 | 24.4 (17.8-34.5) | 11.2-66.7 | 1.0-161.0 |  | 258 | 5.1 (0.8-11.1) | 0-45.4 | 0-158.0 |  |
| Current eczema |  |  |  |  |  |  |  |  |  |  |
| Male | 57 | 42.5 (30.3-52.3) | 20.5-96.7 | 17.1-133.0 | **<0.001** | 31 | 13.9 (5.1-43.6) | 0-179.4 | 0-243 | **0.005** |
| Female | 91 | 27.5 (20.6-35.1) | 10.8-54.7 | 7.6-88.9 |  | 43 | 5.3 (2.0-12.7) | 0-36.4 | 0-63.3 |  |
| Without current eczema |  |  |  |  |  |  |  |  |  |  |
| Male | 459 | 33.0 (23.9-44.9) | 14.2-78.0 | 5.6-346.5 | **<0.001** | 209 | 8.7 (4.2-17.3) | 0-54.9 | 0-196 | **<0.001** |
| Female | 500 | 24.2 (17.7-34.5) | 10.9-66.8 | 1.0-161.0 |  | 232 | 5.1 (0.7-10.7) | 0-47.8 | 0-158.0 |  |
| Atopy |  | | |  |  |  |  | | |  |
| Male | 160 | 36.0 (26.2-50.1) | 15.4-95.1 | 5.6-346.5 | **<0.001** | 94 | 12.1 (6.3-26.4) | 2.0-71.4 | 0-196 | **0.005** |
| Female | 159 | 26.9 (20.2-39.5) | 10.9-70.9 | 7.6-122.0 |  | 75 | 7.1 (3.2-16.7) | 0-73.8 | 0-158 |  |
| Without atopy |  | | |  |  |  |  | | |  |
| Male | 179 | 32.5 (24.1-42.8) | 14.9-71.2 | 7.9-136.0 | **<0.001** | 80 | 7.3 (3.0-13.3) | 0-47.4 | 0-60.7 | **0.010** |
| Female | 283 | 23.5 (16.5-33.8) | 10.4-63.1 | 1.0-122.0 |  | 136 | 4.1 (0-10.3) | 0-34.1 | 0-95.4 |  |
| With asthma, any allergic disease or atopy |  | | |  |  |  |  | | |  |
| Male | 221 | 35.8 (26.8-50.3) | 17.8-95.0 | 5.6-346.6 | **<0.001** | 124 | 10.4 (5.2-22.1) | 0-65.8 | 0-243 | **<0.001** |
| Female | 255 | 26.9 (19.7-35.5) | 11.1-68.6 | 7.6-122.0 |  | 121 | 6.1 (2.4-13.2) | 0-68 | 0-158 |  |
| Without asthma, any allergic disease or atopy |  | | |  |  |  |  |  |  |  |
| Male | 147 | 31.5 (23.9-42.8) | 14.2-75.8 | 7.9-136.0 | **<0.001** | 65 | 7.5 (3.3-13.8) | 0-48.9 | 0-60.7 | **0.011** |
| Female | 213 | 23.2 (16.5-33.9) | 10.4-61.1 | 1.0-122.0 |  | 102 | 4.5 (0-10.5) | 0-42.8 | 0-95.4 |  |

**Details.** Mann-Whitney-U test was performed to compare EDN levels in males and females within each category. Presence of asthma, any allergic disease or atopy was defined based on presence of at least one of the following: current asthma, allergic rhinitis, current eczema, or atopy (defined by at least one skin prick positivity to aeroallergens). **Abbreviations.** EDN= eosinophil-derived neurotoxin, NLF= Nasal lavage fluid, P= percentile, Q= quartile.

**Table E4.** Blood eosinophil count and FeNO levels stratified by sex in different study subpopulations.

|  | **Random adult population**  **Median (Q1-Q3)** | | | **High serum EDN (>75^th^ percentile)**  **Median (Q1-Q3)** | | | **Low serum EDN (≤75^th^ percentile)**  **Median (Q1-Q3)** | | |
| --- | --- | --- | --- | --- | --- | --- | --- | --- | --- |
|  | **Males** | **Females** | ***p*-value** | **Males** | **Females** | ***p*-value** | **Males** | **Females** | ***p*-value** |
| **Blood eosinophil count (cell/mm^3^)** | 200 (100-230) | 190 (100-200) | **0.007** | 300 (200-400) | 300 (200-400) | 0.770 | 100 (100-200) | 100 (90-200) | 0.709 |
| **FeNO (ppb)** | 20.5 (15.1-28.0) | 14.1 (10.8-19.3) | **<0.001** | 25.0 (17.0-38.8) | 20.0 (12.0-30.0) | **<0.001** | 19.7 (14.7-27.7) | 14.8 (11.0-20.1) | **<0.001** |
|  | **Subjects with current asthma**  **Median (Q1-Q3)** | | | **High NLF EDN (>75^th^ percentile)**  **Median (Q1-Q3)** | | | **Low NLF EDN (≤75^th^ percentile)**  **Median (Q1-Q3)** | | |
|  | **Males** | **Females** | ***p*-value** | **Males** | **Females** | ***p*-value** | **Males** | **Females** | ***p*-value** |
| **Blood eosinophil count (cell/mm^3^)** | 200 (100-300) | 200 (100-300) | **0.005** | 200 (100-300) | 200 (200-300) | 0.604 | 200 (100-200) | 200 (100-200) | 0.180 |
| **FeNO (ppb)** | 24.5 (16.0-40.8) | 17.2 (12.0-26.0) | **<0.001** | 25.2 (17.3-35.6) | 18.2 (12.8-25.9) | **<0.001** | 19.3 (13.7-27.7) | 13.9 (10.4-20.9) | **<0.001** |

**Details.** Mann-Whitney-U test was performed to compare blood eosinophil count and FeNO levels in males and females within each category**. Abbreviations.** EDN= eosinophil-derived neurotoxin, FeNO= fractional exhaled nitric oxide, NLF= Nasal lavage fluid, Q= quartile.

1. **Supplementary Figures**

**Figure E1.** Flow chart of the study participants with serum EDN levels (n = 2,939). **Details.** *Since 22 cases participated more than one visit, latest visit was excluded in case of multiple measurements. **Abbreviations.** EDN= Eosinophil-derived neurotoxin.

**Figure E2.** Flow chart of the study participants with nasal lavage fluid EDN levels (n = 878). **Details.** *Subjects without NLF cell count was also not included. **Abbreviations.** EDN= Eosinophil-derived neurotoxin, NLF= Nasal lavage fluid.

1. **Serum EDN levels B. NLF EDN levels**


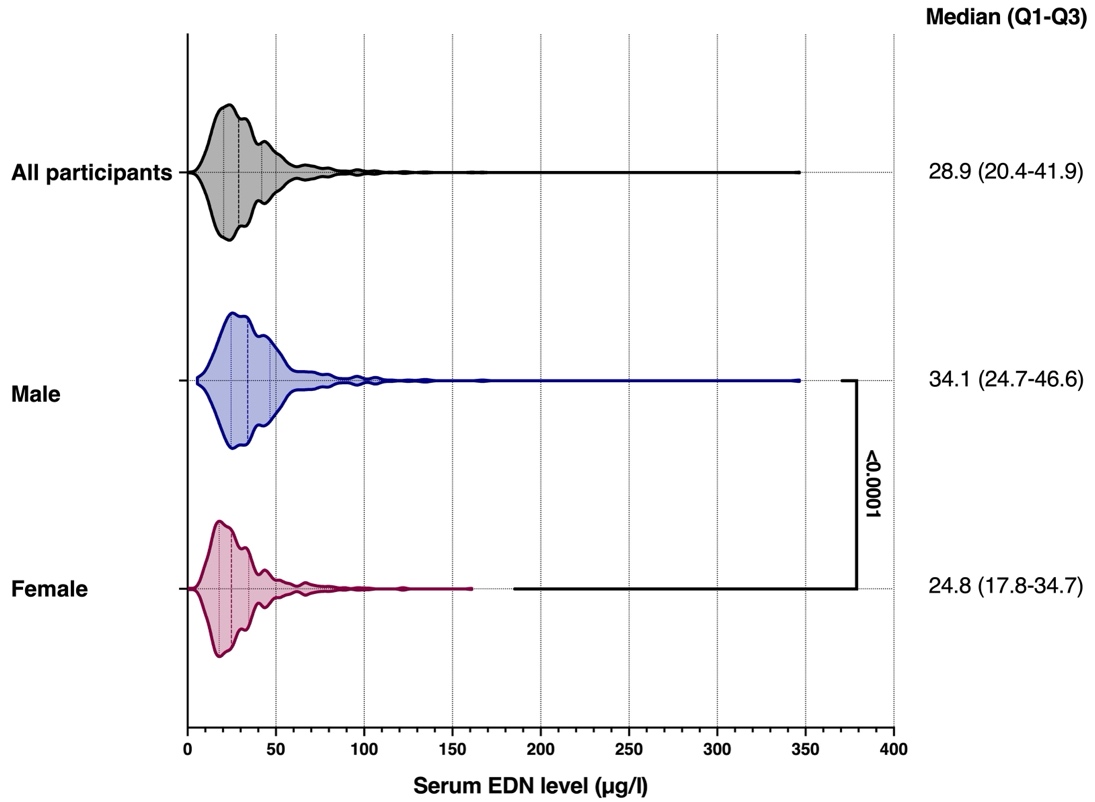

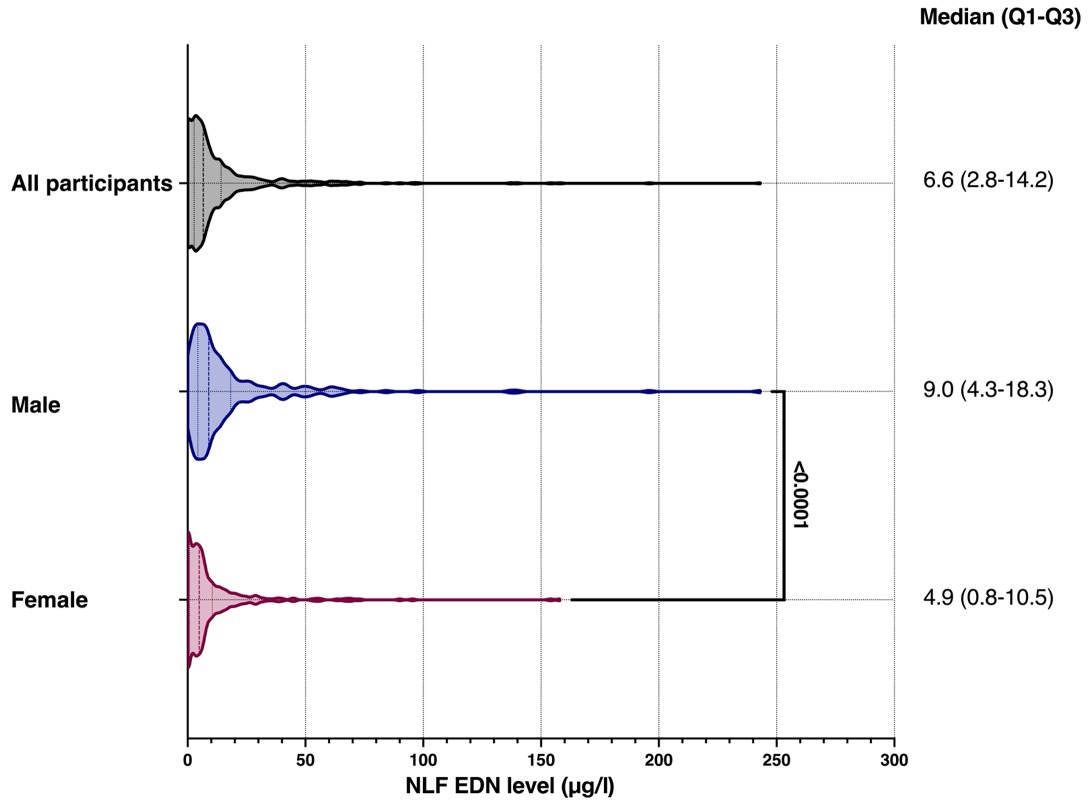


**Figure E3.** Violin plot of serum and nasal lavage fluid EDN levels in random adult population stratified by sex (truncated). **Abbreviations.** EDN= Eosinophil-derived neurotoxin, NLF=Nasal lavage fluid, Q=quartile.

1. **Current asthma vs. without current asthma B. Current rhinitis vs. without current rhinitis**

**Figure E4.** Receiver operating characteristic curve for serum EDN level (µg/l) in current asthma vs without current asthma **(Figure E4A)** and current rhinitis vs without current rhinitis **(Figure E4B**). **Details.** Threshold values calculated based on Youden index. **Abbreviations.** AUC= Area under curve, EDN= Eosinophil-derived neurotoxin.

1. **Current asthma vs. without current asthma B. Current rhinitis vs. without current rhinitis**

**Figure E5.** Receiver operating characteristic curve for nasal lavage fluid EDN level (µg/l) in current asthma vs without current asthma **(Figure E5A)** and current rhinitis vs without current rhinitis **(Figure E5B).** **Details.** Threshold values calculated based on Youden index. **Abbreviations.** AUC= Area under curve, EDN= Eosinophil-derived neurotoxin.

1. **Current asthma vs. without current asthma in males B. Current rhinitis vs. without current rhinitis in males**

**Figure E6.** Receiver operating characteristic curve for serum EDN level (µg/l) in current asthma vs without current asthma **(Figure E6A)** and current rhinitis vs without current rhinitis in males **(Figure E6B**). **Details.** Threshold values calculated based on Youden index. **Abbreviations.** AUC= Area under curve, EDN= Eosinophil-derived neurotoxin.

1. **Current asthma vs. without current asthma in males B. Current rhinitis vs. without current rhinitis in males**

**Figure E7.** Receiver operating characteristic curve for nasal lavage fluid EDN level (µg/l) in current asthma vs without current asthma **(Figure E7A)** and current rhinitis vs without current rhinitis in males **(Figure E7B).** **Details.** Threshold values calculated based on Youden index. **Abbreviations.** AUC= Area under curve, EDN= Eosinophil-derived neurotoxin.

1. **Current asthma vs. without current asthma in females B. Current rhinitis vs. without current rhinitis in females**

**Figure E8.** Receiver operating characteristic curve for serum EDN level (µg/l) in current asthma vs without current asthma **(Figure E8A)** and current rhinitis vs without current rhinitis in females **(Figure E8B**). **Details.** Threshold values calculated based on Youden index. **Abbreviations.** AUC= Area under curve, EDN= Eosinophil-derived neurotoxin.

1. **Current asthma vs. without current asthma in females B. Current rhinitis vs. without current rhinitis in females**

**Figure E9.** Receiver operating characteristic curve for nasal lavage fluid EDN level (µg/l) in current asthma vs without current asthma **(Figure E9A)** and current rhinitis vs without current rhinitis in females **(Figure E9B).** **Details.** Threshold values calculated based on Youden index. **Abbreviations.** AUC= Area under curve, EDN= Eosinophil-derived neurotoxin.


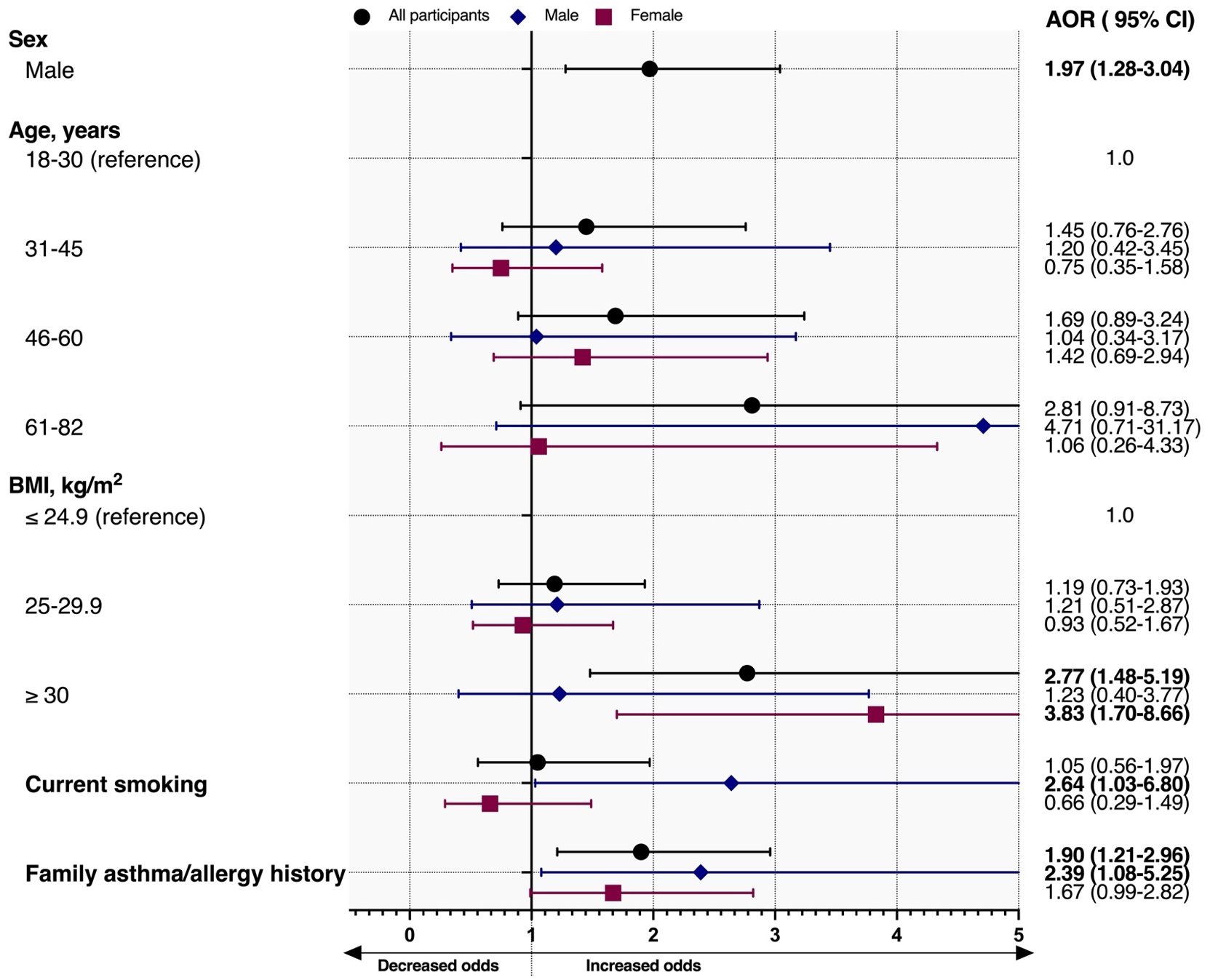


**Figure E10.** Determinants of high serum EDN levels (above 75^th^ percentile) stratified by sex in subjects without asthma, any allergic disease, or atopy. **Details.** Results were adjusted for sex, age, current smoking, BMI, family history of asthma/allergy. Sex-specific models were adjusted for age, current smoking, BMI, family history of asthma/allergy. Whiskers demonstrate upper and lower limits of confidence intervals. **Abbreviations.** AOR= Adjusted odds ratio, BMI= Body mass index, CI= Confidence interval.

**
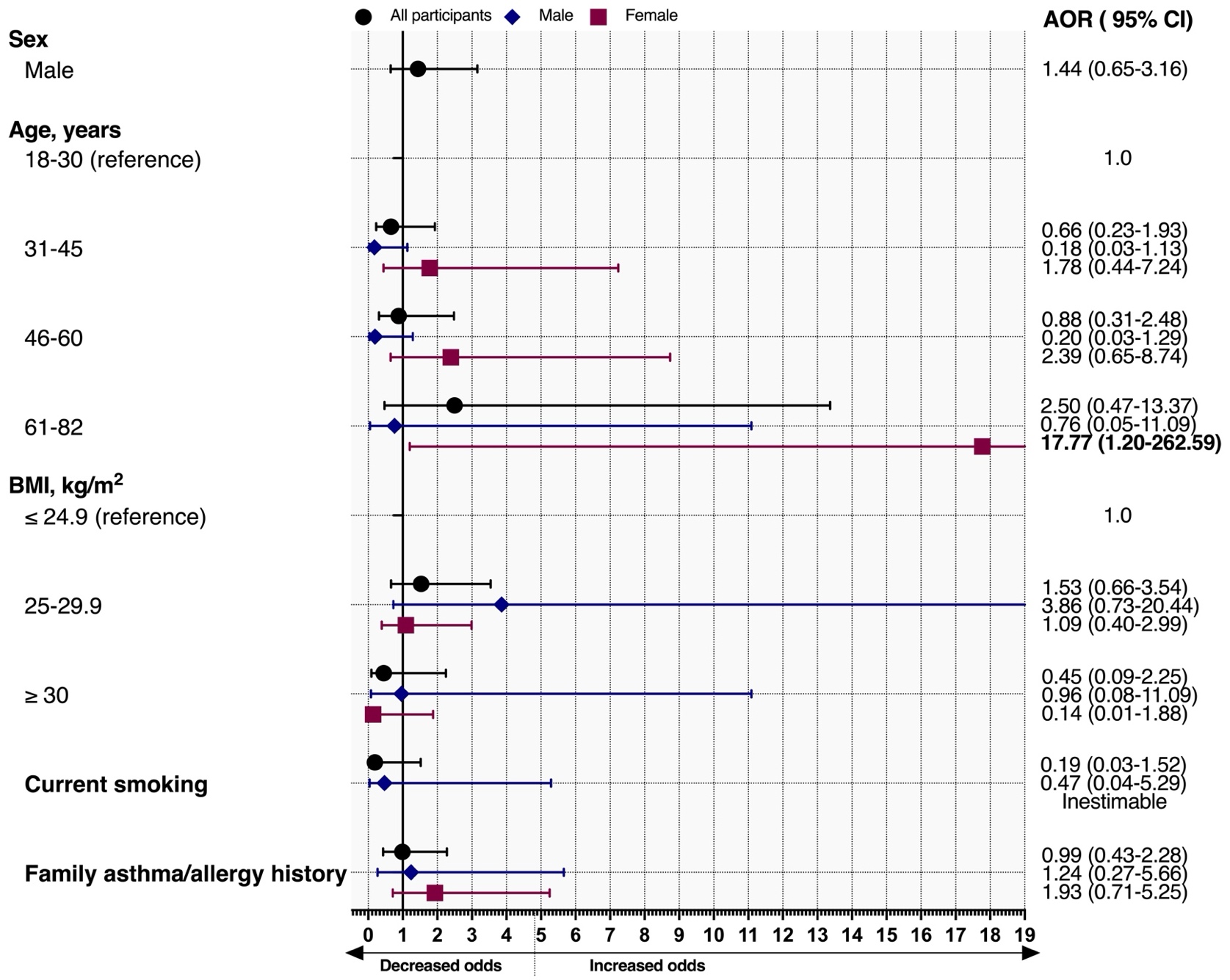
**

**Figure E11.** Determinants of high NLF EDN levels (above 75^th^ percentile) stratified by sex in subjects without asthma, any allergic disease, or atopy. **Details.** Results were adjusted for sex, age, current smoking, BMI, family history of asthma/allergy. Sex-specific models were adjusted for age, current smoking, BMI, family history of asthma/allergy. Whiskers demonstrate upper and lower limits of confidence intervals. **Abbreviations.** AOR= Adjusted odds ratio, BMI= Body mass index, CI= Confidence interval.


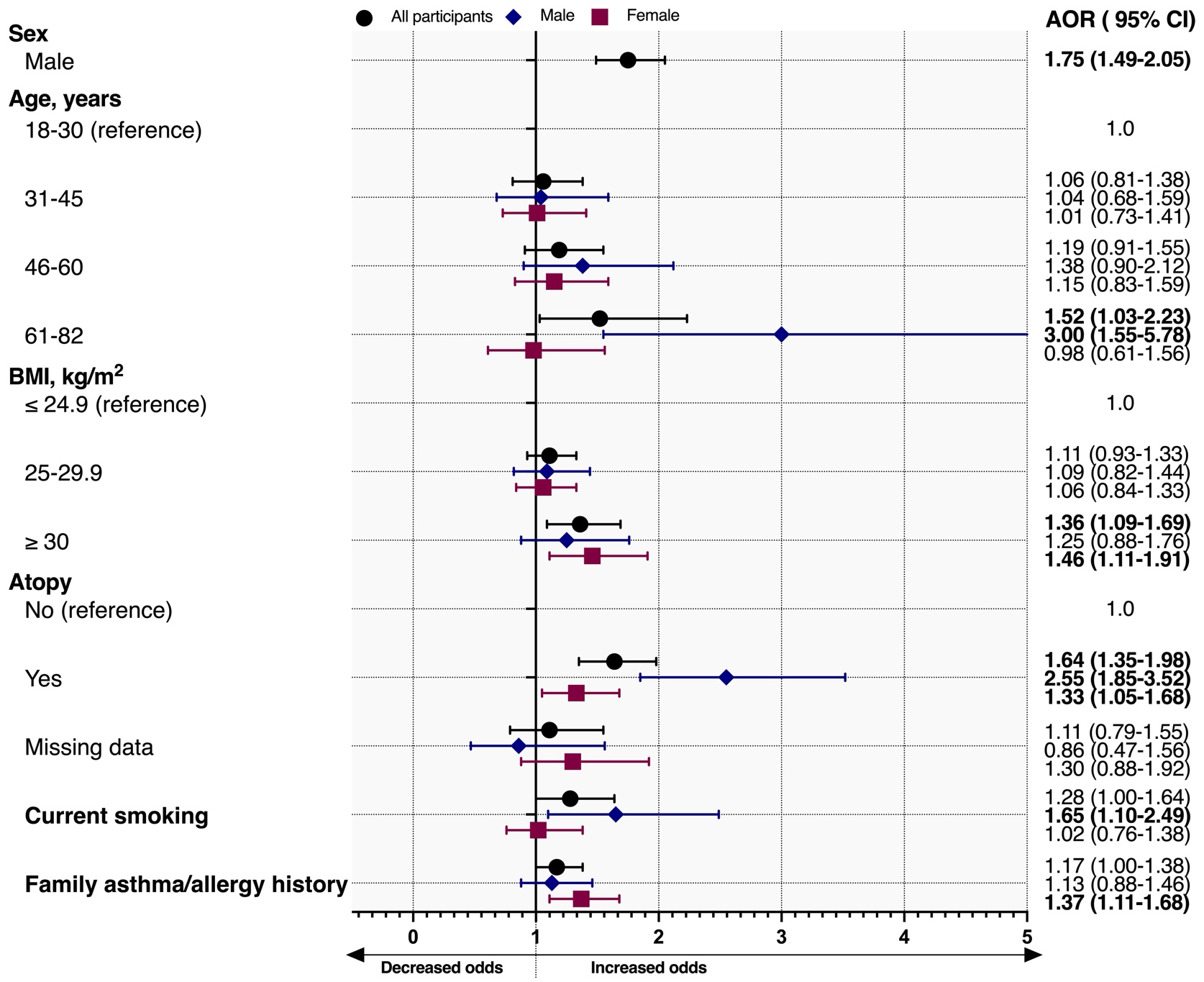


**Figure E12.** Determinants of high serum EDN levels (above 75^th^ percentile) stratified by sex in all participants. **Details.** Results were adjusted for sex, age, current smoking, BMI, family history of asthma/allergy, and atopy. Sex-specific models were adjusted for age, current smoking, BMI, family history of asthma/allergy, and atopy. Whiskers demonstrate upper and lower limits of confidence intervals. **Abbreviations.** AOR= Adjusted odds ratio, BMI= Body mass index, CI= Confidence interval.


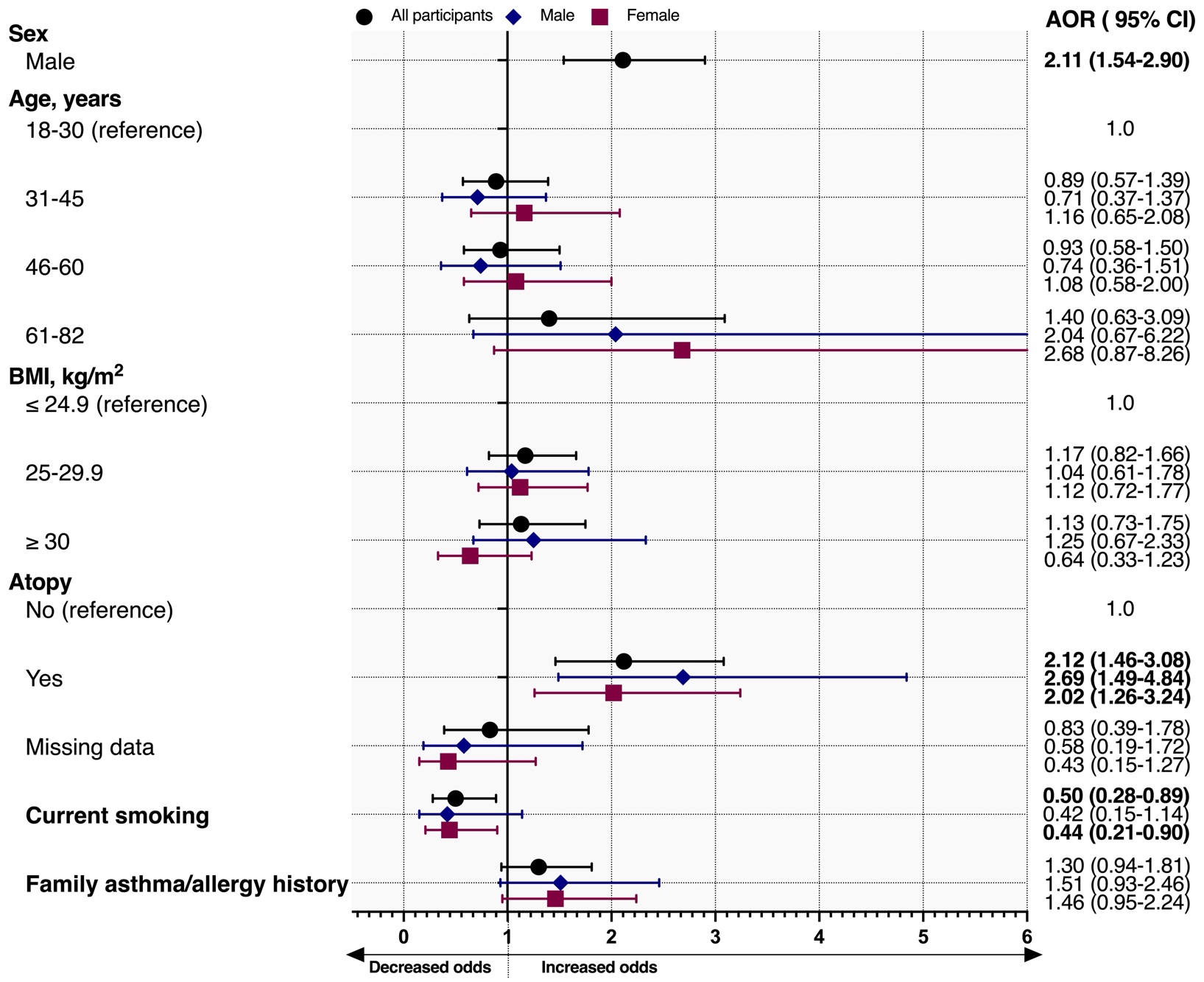


**Figure E13**. Determinants of high NLF EDN levels (above 75^th^ percentile) stratified by sex in all participants. **Details.** Results were adjusted for sex, age, current smoking, BMI, family history of asthma/allergy, and atopy. Sex-specific models were adjusted for age, current smoking, BMI, family history of asthma/allergy, and atopy. Whiskers demonstrate upper and lower limits of confidence intervals. **Abbreviations.** AOR= Adjusted odds ratio, BMI= Body mass index, CI= Confidence interval.

1. **Correlation matrix in random sample, males B. Correlation matrix in random sample, females**

**C. Correlation matrix in subjects with current asthma, males D. Correlation matrix in subjects with current asthma, females**

**Figure E14**. Correlation matrix (Spearman’s rho) between serum EDN levels, NLF EDN levels, blood eosinophil count, and FeNO levels stratified in males in random adult population and in those with current asthma. **Details.** Significant correlations were marked with asterisks. Abbreviations. EDN= Eosinophil-derived neurotoxin, FeNO= Fractional exhaled nitric oxide, NLF = Nasal lavage fluid.

**A. Serum EDN level and blood eosinophil count B. Serum EDN level and FeNO levels C. NLF EDN level and blood eosinophil count**

**D. NLF EDN level and FeNO level E. Serum EDN and NLF EDN level**

**Figure E15.** Correlation (Spearman’s rho) in the random adult population between serum EDN level (µg/l) and blood eosinophil count **(A)**; serum EDN level and FeNO level (ppb) **(B)**; NLF EDN level (µg/l) and blood eosinophil count **(C)**; NLF EDN levels and FeNO levels **(D)**; correlation between serum EDN levels and NLF EDN levels **(E).** **Abbreviations.** EDN= Eosinophil-derived neurotoxin, FeNO= Fractional exhaled nitric oxide, NLF = Nasal lavage fluid.
